# Regulation of Semaphorin3A in the process of cutaneous wound healing

**DOI:** 10.1038/s41418-022-00981-6

**Published:** 2022-03-26

**Authors:** Yang Zheng, Feng Jiang, Chao Wang, Mengjie Dong, Chundi Wang, Enshi Yan, Yi Wang, Zaiou Zhu, Xianbin Xiong, Xu Ding, Jinhai Ye, Yue He, Hongchuang Zhang, Junbo Zhou, Wei Zhang, Yunong Wu, Xiaomeng Song

**Affiliations:** 1 Jiangsu Key Laboratory of Oral Diseases, Nanjing Medical University, Nanjing, Jiangsu PR China; 2 Department of Oral Maxillofacial & Head and Neck Oncology, Shanghai Ninth People’s Hospital Affiliated to Shanghai Jiao Tong University School of Medicine, National Clinical Research Center of Stomatology, Shanghai, PR China; 3 Department of Oral and Maxillofacial Surgery, Affiliated Hospital of Stomatology, Nanjing Medical University, Nanjing, Jiangsu PR China; 4 Jiangsu Province Engineering Research Center of Stomatological Translational Medicine, Nanjing Medical University, Nanjing, Jiangsu PR China; 5 Department of Stomatology, Fuyang Hospital of Anhui Medical University, Fuyang, Anhui PR China; 6 Department of Anesthesiology, Affiliated Stomatological Hospital, Nanjing Medical University, Nanjing, Jiangsu PR China; 7 Department of Stomatology, Xuzhou No. 1 Peoples Hospital, Xuzhou, Jiangsu PR China; 8 Department of Stomatology, Nanjing Integrated Traditional Chinese and Western Medicine Hospital, Nanjing, Jiangsu PR China

**Keywords:** Gene regulation, Experimental models of disease

## Abstract

Semaphorin 3A (Sema3A) has been recognized as a crucial regulator of morphogenesis and homeostasis over a wide range of organ systems. However, its function in cutaneous wound healing is poorly understood. In our study, we demonstrated that Sema3A adenovirus plasmids transfection limited keratinocyte proliferation and decreased migrative capacity as assessed by in vitro wound healing assay. Sema3A transduction inhibited TGF-β1-mediated keratinocyte migration and EMT process. Besides, we applied mice with K14-Cre-mediated deletion of Sema3A and found that Sema3A depletion postponed wound closure with decreased re-epithelialization and matrix growth. Contrary to the results obtained with full-length Sema3A plasmids transfection, increased keratinocyte migration with recombinant Sema3A proteins resulted in quicker closure of the wounding area after a scratch. Further, exogenously applied recombinant Sema3A worked with EGF to maintain the activation of EGFR by interacting with NRP1 and thereby regulated the internalization of the EGFR-NRP1 complex. Taken together, these results indicated a paradoxical role of autonomous and non-autonomous Sema3A expression during wound healing. Combined administration of recombinant EGF and Sema3A proteins could accelerate the process of wound repair, thus providing promising treatment prospects in the future.

## Introduction

The process of wound re-epithelialization requires efficient coordination of multiple events that involve hemostasis, inflammation, proliferation, re-epithelialization and remodeling of new epithelium into stratified epidermis [1, 2]. Following the initial formation of the wound bed, basal and suprabasal keratinocytes proliferate and migrate to fill the wound and reestablish the first layer of new epithelium [3]. Keratinocyte migration, which is required for re-epithelialization during the proliferation phase, is the most limiting step and is tightly regulated by various signaling pathways [4]. However, the factors that regulate keratinocyte mobilization in response to wound healing have not been well established [5, 6].

To migrate over the wound site, keratinocytes at the wound site undergo the epithelial-mesenchymal transition (EMT) process, a phenotype shift from adherent epithelial cells to a more dynamic state [7, 8]. This process of EMT allows polarized epithelial cells that are in contact with a basement membrane to acquire a motile mesenchymal state related to enhanced motility and increased production of extracellular matrix (ECM) proteins [9]. EMT is usually characterized by the alteration of associated proteins and inducers involving decreased epithelial markers, including E-cadherin and β-catenin, and increased mesenchymal markers, such as N-cadherin, vimentin, snail and ZEB2 [10–12]. TGF-β1 has been identified as an important inducer of EMT in normal human keratinocytes and cutaneous wound repair [13]. Briefly, activation of TGF-β1 leads to phosphorylation of its downstream signaling molecules Smad2/3, which in turn induces target genes involved in cell proliferation and migration [14, 15]. Although EMT has been implicated in the process of wound healing, there are limited data on the cytokines and molecules regulating cutaneous EMT.

Semaphorin 3A (Sema3A) is an extracellular matrix molecule that inhibits axonal outgrowth and causes growth cone collapse via the receptor complex formed by Neuropilin-1 (NRP1), four type-A plexins and plexin-D1 [15, 16]. NRP1 is a single-pass transmembrane receptor that is involved in multiple physiological and pathological processes [17]. NRP1 provides a binding site for Sema3A, while members of the plexin family transduce Sema3A signals into cells through its cytoplasmic domain [18]. NRP1 also serves as a receptor for some members of the vascular endothelial growth factor (VEGF) family [19, 20]. In endothelial cells, Sema3A competes with VEGF_165_ and inhibits cell motility [21]. Although the function of Sema3A/NRP1 in neural development has been studied intensively, the fact that the complex is expressed in many different tissues suggests that it also plays a role in other organs. Sema3A is increasingly being recognized as a key suppressor of tumor growth and metastasis [22, 23]. Rb-Sema3A greatly potentiated TGF-β1-induced profibrotic effects and helped regulate epithelial corneal wound healing [24]. Our group has previously identified the tumor-suppressive role of Sema3A in head and neck cancer cells [22]. However, the function of Sema3A in skin regeneration and functional recovery after wound healing has not been fully studied. In keratinocytes, of the two NRP1 ligands, VEGF_165_ does not affect keratinocyte characteristics, whereas Sema3A inhibits migration. Sema3A might also modulate epidermal innervation in atopic dermatitis [25].

Epidermal growth factor receptors (EGFRs) are intrinsic tyrosine kinase receptors expressed in the cell membranes of normal tissues in the salivary glands, skin, etc [26]. Epidermal growth factor (EGF) binds to EGFR and plays an exceedingly important role in wound healing by regulating the migration, proliferation, morphology and plasticity of epithelial cells [27]. At the wound site, EGF stimulates wound closure. High levels of EGFR-dependent autocrine extracellular signal-regulated kinase (ERK) activation have been observed previously in keratinocytes [28]. In oral keratinocytes, EGF increases ERK1/2 phosphorylation of the linker region of the transcription factor Smad2 to regulate the duration of TGF-β1 signaling [29]. EGFR/ERK signaling increased the expression of angiogenesis-associated genes, resulting in enhanced re-epithelialization, angiogenesis, and wound healing.

In our study, we demonstrated that Sema3A plasmids transfection negatively regulated wound healing. In addition, we utilized mice with K14-Cre-mediated deletion of Sema3A and found that Sema3A depletion postponed wound closure with decreased re-epithelialization and matrix growth. Contrary to the results obtained with Sema3A transfection, increased keratinocyte migration with recombinant Sema3A protein resulted in quicker closure of the wound area after a scratch. Mechanistically, NRP1 interacted with and mediated EGFR internalization from the cell surface, leading to activation of intracellular signaling. Exogenously applied Sema3A proteins combined with EGF could maintain the activation of EGFR and NRP1, thus providing promising treatment prospects in the future.

## Results

### Sema3A-overexpressing adenovirus plasmids contributed to decreased keratinocyte migration and proliferation

**Fig. 1:**
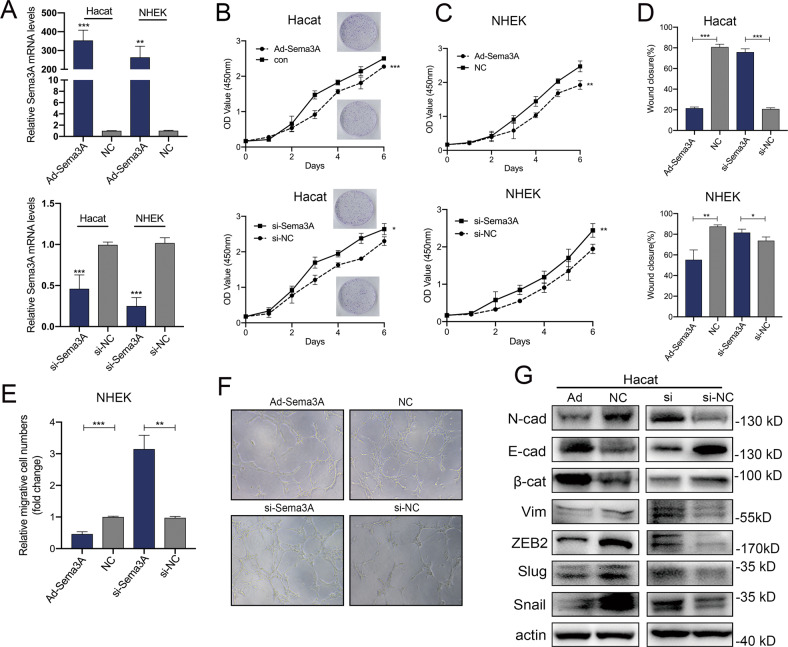
Sema3A-overexpressing adenovirus plasmids contributed to decreased keratinocyte migration and proliferation. **A** Transfection efficiency was confirmed by qRT-PCR analysis in Hacat and NHEK cells. Bars indicate the mean fold changes ± SEM relative to the control; *n* = 4. **B** The effect of Sema3A adenovirus plasmids on the proliferation potential of Hacat cells was analysed by CCK-8 and Colony formation experiments. Data are shown as means ± SEM; *n* = 4. **C** The effect of Ad-Sema3A on the proliferation potential of NHEK cells was analysed by CCK-8. Data are shown as means ± SEM; *n* = 4. **D** Wound healing assays were performed in Ad-Sema3A or si-Sema3A-transfected Hacat and NHEK cells. The percentage of wound closure is displayed as the mean ± SEM; *n* = 3. **E** Transwell assays showed that transfection with adenovirus Seam3A restrained the migratory ability, while Sema3A inhibition reversed this effect. Bars indicate the mean fold changes ± SEM relative to the corresponding control; *n* = 3. **F** Angiogenesis in HUVEC during the incubation with supernatant gathered from Sema3A- or si-Sema3A-transfected keratinocytes. **G** Western blotting analysis of EMT markers in Hacat cells transfected with Ad-Sema3A, si-Sema3A and the relative control. *P < 0.05; **P < 0.01; ***P < 0.001.

First, to determine the biological function of Sema3A in keratinocytes, NHEKs and Hacat cells were transfected with Sema3A-overexpressing adenovirus or RNAi. The transfection efficiency was determined by qRT-PCR (Fig. 1A). CCK-8 and colony formation assays showed that inhibition of Sema3A significantly enhanced the viability of keratinocytes, while the overexpression of Sema3A showed the opposite effects (Fig. 1B, C). Additionally, the migration and invasion rates of keratinocytes were noticeably decreased due to Sema3A overexpression (Fig. 1D, E). In contrast, Sema3A suppression stimulated the migration and invasion of cells (Fig. 1D, E). Meanwhile, tube formation assay confirmed the antiangiogenic effect of Sema3A in HUVECs (Fig. 1F). To further seek alternative mechanisms beyond the repression of Sema3A, we turned to the mutual interaction between endogenous Sema3A and EMT. To address the role of Sema3A in this process, EMT-related markers were assessed by immunoblot analysis. Sema3A reduced the expression of a myriad of mesenchymal markers (N-cadherin, Vimentin, Snail, Slug and ZEB2) and upregulated the expression of the epithelial marker E-cadherin and β-catenin. In contrast, upregulation of mesenchymal markers was associated with Sema3A inhibition (Fig. 1G).

### Sema3A transduction suppressed TGF-β1-induced keratinocyte migration in a NRP1-dependent manner

**Fig. 2:**
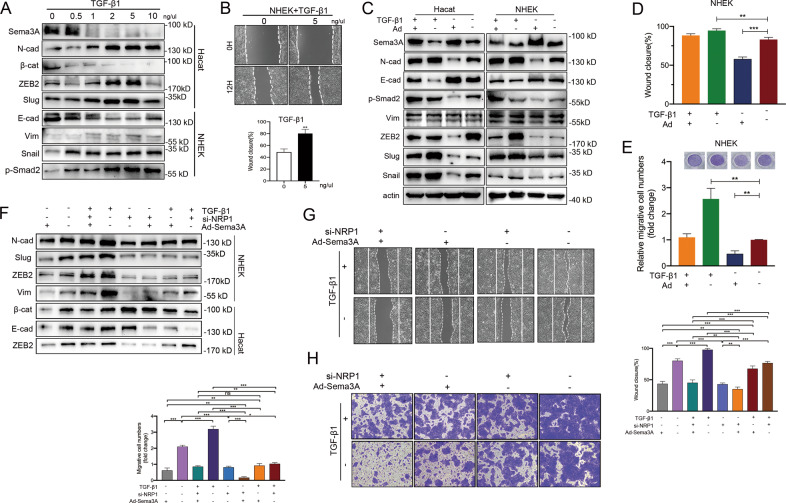
Sema3A transfection suppressed TGF-β1-induced keratinocyte migration in a NRP1-dependent manner. **A** Western blotting analysis of Sema3A and EMT markers after exposure to escalated concentrations of TGF-β1 in Hacat and NHEK cells. **B** Wound healing experiment of incubation with TGF-β1. Data are shown as means ± SEM; *n* = 3. **C** Sema3A adenovirus plasmids were transfected into keratinocytes in the absence or presence of TGF-β1. The expression of EMT markers and the phosphorylation of Smad2/3 were shown by western blotting. Wound healing (**D**) and Transwell (**E**) assays in transfected Ad-Sema3A keratinocytes in the absence or presence of TGF-β1. The percentage of wound closure is displayed as the mean ± SEM; *n* = 3. For the transwell assays, bars indicate the mean fold changes ± SEM relative to the corresponding control; *n* = 3. **F** EMT-related proteins were determined in Ad-Sema3A ± si-NRP1-transfected cells with or without TGF-β1. Phenotypic alterations were verified by wound healing (**G**) and transwell (**H**) assays. *P < 0.05; **P < 0.01; ***P < 0.001.

Members of the transforming growth factor-β (TGF-β) superfamily have been identified as important inducers of EMT. We then evaluated the level of Sema3A during TGF-β1 exposure in keratinocytes. We observed an obvious alteration in Sema3A protein expression and migrative capacity of NHEK cells due to TGF-β1 stimulation (Fig. 2A, B). To investigate whether Sema3A may be involved in wound healing-mediated EMT, we transfected Sema3A-overexpressing adenovirus into keratinocytes in the absence or presence of TGF-β1 and determined the expression of EMT markers and the phosphorylation of Smad2/3. Keratinocytes transfected with Sema3A had reduced expression of p-Smad2, N-cadherin, Snail, slug and ZEB2 as well as a slight induction of E-cadherin upon TGF-β1 stimulation (Fig. 2C), indicating that Sema3A was involved in the EMT process by TGF-β1. By performing wound healing and Transwell assays, we discovered that Sema3A expression partially reversed the migration effect when TGF-β1 was introduced, demonstrating that one of the principle mechanisms through which Sema3A affects wound closure in vitro is due to the interaction with TGF-β1 (Fig. 2D, E). Whereas NRP1 has been considered as a major component of the Sema3A receptor complex, we elicited the potential role of NRP1 during this process. We then examined the effects of NRP1 on Sema3A-induced EMT phenotype alterations. Upon upregulation of Sema3A with the deletion of NRP1, the migrative capacity of keratinocytes decreased (Fig. 2G, H). In the presence of TGF-β1, when transfected with anti-NRP1 siRNA, Sema3A-transfected cells retained epithelial morphology. Thus, EMT markers verified the morphologic changes (Fig. 2F). To conclude, these results indicated that Sema3A-induced EMT alteration depended on the expression of NRP1 in keratinocytes.

### Ad-Sema3A transfection suppressed activation of EGFR/ERK axis

**Fig. 3:**
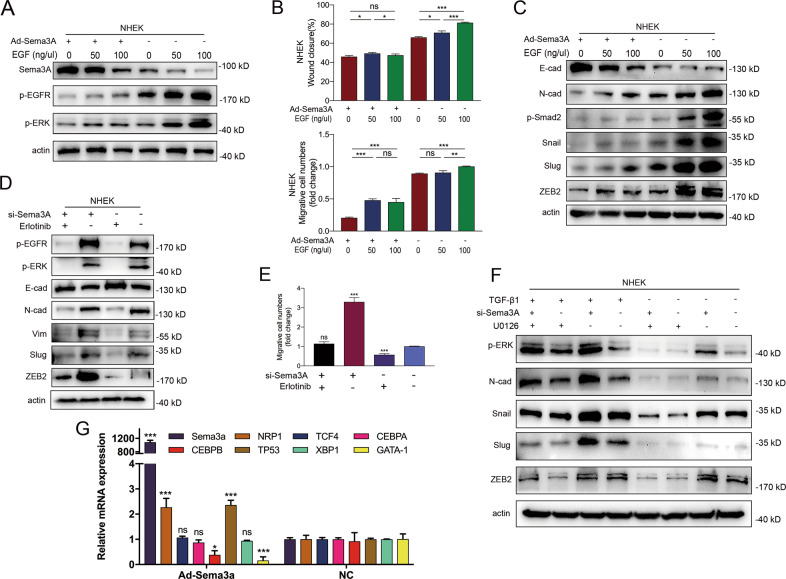
Ad-Sema3A transfection suppressed activation of EGFR/ERK axis. **A** Ad-Seam3A plasmids were transfected into NHEK cells for 48 h. Then, recombinant EGF protein was added to the transfected cells for 15 min. Sema3A, p-EGFR and p-ERK were analysed by western blot. **B** Wound healing and Transwell assays in transfected Sema3A plasmids in the absence or presence of EGF. **C** Recombinant EGF protein was incubated in the Ad-Sema3A- or NC-transfected cells for 2 days, and immunoblotting analysis is displayed. **D** Transfection of short peptides interfering with Sema3A function in keratinocytes, treatment with the EGFR signal inhibitor erlotinib, and testing of the protein expression of EMT markers. **E** Cells treated with si-Sema3A ± erlotinib were plated in the chamber, and the migration capacity was assessed. Bars indicate the fold changes ± SEM relative to the negative control. **F** The ERK-specific inhibitor U0126 was introduced into NHEKs. Western blot analysis showed that U0126 attenuated the EMT process mediated by TGF-β1 and that Sema3A deficiency enhanced the protein expression of mesenchymal markers triggered by U0126. **G** qRT-PCR analysis was performed to confirm the expression level of transcriptional factors including Sema3A, NRP1, GATA-1, CEBPA, XBP1, TP53, CEBPB and TCF4 in Hacat cells. *P < 0.05; **P < 0.01; ***P < 0.001.

Epidermal growth factor receptor (EGFR) can be activated by a family of ligands that include epidermal growth factor (EGF). This binding event causes trans-autophosphorylation of EGFR, resulting in signaling cascades of Ras, Raf, MEK, and extracellular signal-regulated kinase (ERK) to induce phosphorylation of myriad proteins critical for cell motility and wound healing [27]. As shown in Fig. S2A, B, EGF stimulated cell proliferation and migration in a dose-dependent manner. Meanwhile, keratinocytes displayed distinct morphological changes characterized principally by an elongated and fibroblast-like phenotype (data not shown). Activation of EGF also upregulated the expression of EGFR-ERK signaling, N-cadherin, vimentin and ZEB2 along with the downregulation of E-cadherin (Fig. S2B), which indicated the induction of EMT by EGF stimulation. To test cell mobility alterations induced by EGF, we treated keratinocytes with various concentrations of EGF with or without the transfection of Ad-Sema3A. Ad-Sema3A effectively inhibited the activation of EGFR-ERK signaling activated by EGF (Fig. 3A) and negated the EMT transition (Fig. 3B, C). It is believed that activation of ERK/MAPK might represent one point of intersection in semaphorin signaling [30]. Next, we transfected short peptides interfering with Sema3A function in keratinocytes, treated them with the EGFR signal inhibitor erlotinib, and tested the protein expression of EMT markers. Erlotinib resulted in the downregulation of N-cadherin, and Sema3A inhibition compromised the suppressive effect caused by erlotinib (Fig. 3D). Similar results were obtained in transwell assays (Fig. 3E). Finally, we utilized the ERK-specific inhibitor U0126 to determine the inhibitory regulation of ERK signal suppression in the EMT process after exposure to TGF-β1 and anti-Sema3A siRNA. U0126 attenuated the EMT process mediated by TGF-β1, and Sema3A deficiency enhanced the protein expression of mesenchymal markers triggered by U0126 (Fig. 3F). These data suggested that the EGFR-ERK pathway contributed to the migration and EMT of keratinocytes and that transfection of Ad-Sema3A could reverse this process. To investigate the potential mechanism that Sema3A transfection regulates EGFR pathway, we performed qRT-PCR in Hacat cells transfected with Sema3A and tested several transcription factors predicted to have binding sites to the promoter sequences of EGFR (Fig. S2C). mRNA levels of CEBPB and GATA1 were significantly decreased after Ad-Sema3A transfection (Fig. 3G). Further experiments can be focused on the two transcription factors when determining the function of Sema3A expression in EGFR-ERK pathway.

### Loss of Sema3A delayed cutaneous wound healing in vivo

**Fig. 4:**
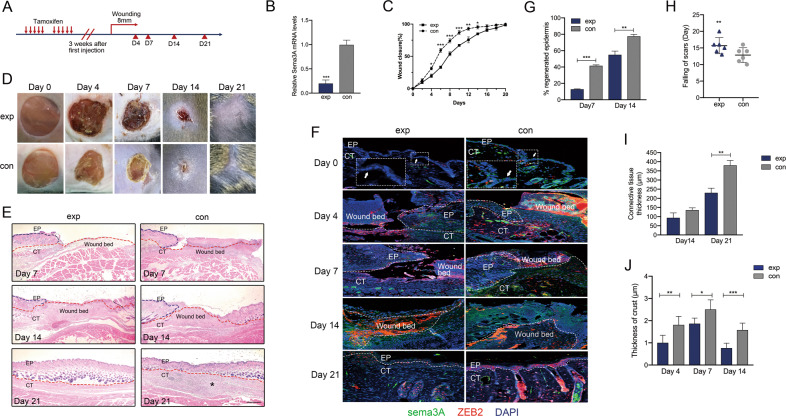
Loss of Sema3A delayed cutaneous wound healing in vivo. **A** Schematic representation of the wound-healing studies performed in K14-Cre^TM+^;*Sema3A*^*L/L*^ and K14-Cre^TM-^;*Sema3A*^*L/L*^ mice. **B** qRT-PCR analysis of epidermal Sema3A mRNA after tamoxifen induction. GAPDH served as control. Bars indicate the mean fold changes ± SEM relative to the control (K14-Cre^TM-^;*Sema3A*^*L/L*^ group); *n* = 3. **C** Quantification of the wound closure area at different time points after wounding in the exp (K14-Cre^TM+^;*Sema3A*^*L/L*^) and con (K14-Cre^TM-^;*Sema3A*^*L/L*^) groups. Data are shown as means ± SEM; *n* = 6. **D** Representative macroscopic illustration of wound healing in exp and con animals at Days 0, 4, 7 and 14. **E** H&E-stained sections of wounds used for morphometric analysis of the percentage of wound closure (length of newly formed epithelium (NFE)/length of NFE + length of gap between edges of wound epithelium (red dotted line) × 100) and re-epithelialization (length of NFE). White asterisk (*) indicates the proliferative connective tissue in the control group. Scale bar = 200 µm. **F** Quantification of the percentage of Sema3A/ZEB2 + area of the epithelial and granulation tissue at different time points. **G** Quantification of the percentage of wound re-epithelialization at Days 7 and 14 after wounding in K14-Cre^TM+^;*Sema3A*^*L/L*^ and K14-Cre^TM-^;*Sema3A*^*L/L*^ wounds. Data are shown as means ± SEM; *n* = 6. **H** Comparison of the healing times (scab falling off) in the days after wounding. Data are shown as means ± SEM; *n* = 6. **I** Comparison of connective tissue in control and *Sema3A* cKO mice. Data are shown as means ± SEM; *n* = 6. **J** Thickness of crust after injury. Bars indicate the mean fold changes relative to con (K14-Cre^TM-^;*Sema3A*^*L/L*^) ± SEM; *n* = 6. *P < 0.05; **P < 0.01; ***P < 0.001.

To further determine the role of Sema3A during in vivo wound healing, we intercrossed tamoxifen-inducible K14-Cre mice with floxed Sema3A mice (Fig. S1) and determined Sema3A expression in epidermis, venous blood and muscular tissues (Figs. 4B and S3A, B). Sema3A expression was decreased by 80% or 50% in the epithelium or venous blood of experimental (K14-Cre^TM+^;*Sema3A*^*L/L*^) mice (Figs. 4A, B and S3A, B). Compared to controls (K14-Cre^TM-^;*Sema3A*^*L/L*^), K14-Cre^TM+^;*Sema3A*^*L/L*^ mice were normal and had similar epidermal features. Full-thickness standardized excisional wounds (8 mm in diameter) were induced in 12-wk-old experimental mice and controls [31, 32]. Pictures were taken daily of the wounds to determine rates of closure. The wound-closure area, quantified at different time points following injury, showed that from Day 4 postinjury, healing was significantly slower in the experimental group than in the control group (Fig. 4C). Representative photographs of dorsal wounds on Days 0, 4, 7, 14 and 21 from K14-Cre^TM+^;*Sema3A*^*L/L*^ mice and K14-Cre^TM-^;*Sema3A*^*L/L*^ mice are shown in Fig. 4D. Haematoxylin and eosin (H&E)-stained sections of K14-Cre^TM+^;*Sema3A*^*L/L*^ mice and K14-Cre^TM-^;*Sema3A*^*L/L*^ mouse wounds on Days 7, 14 and 21 postinjury are visualized in Fig. 4E (Scale bar, 200 μm). Existing evidence supports the functional involvement of ZEB2 in cutaneous wound healing. We utilized immunofluorescence at the indicated time points during wound healing (Fig. 4F). Electron microscopy analyses further indicated that Sema3A accumulated at the surface of the dorsal epidermis in the control group on Day 0. After excision, epithelial tissue proliferated rapidly in the control group along with Sema3A expression. The wound bed displayed extensive expression of Sema3A and ZEB2 coexpression by Day 4. Meanwhile, K14-Cre^TM+^;*Sema3A*^*L/L*^ mice showed negative Sema3A/ZEB2 staining. The K14-Cre^TM-^;*Sema3A*^*L/L*^ group grew more rapidly and reached confluence faster than the K14-Cre^TM+^;*Sema3A*^*L/L*^ keratinocyte group (Day 7). On Day 21, the control group mice displayed a thicker epidermis than their littermates with weaker ZEB2 immunostaining. Re-epithelialization was then measured by determining the ratio of the distance between the epithelial edges and the edges of the wound (Fig. 4G). Analysis of wound morphology 7 d after injury revealed epidermal healing of more than 41% re-epithelialization of the wounds in K14-Cre^TM-^;*Sema3A*^*L/L*^ mice compared with 13% in the K14-Cre^TM+^;*Sema3A*^*L/L*^ group (Fig. 4G). Comparison of the healing times (scab falling off) was analysed in the days after wounding (Fig. 4H). The thicknesses of connective tissues were measured and were shown in Fig. 4I. The control group showed increased proliferation in the connective tissues under the dermal layer as a result of the high proliferative activity of fibroblasts (Fig. 4F, I). Furthermore, we observed greater infiltration in the experimental group and severe inflammation around the wound. The thickness of the crust demonstrated the condition of leakage of serum proteins on the wound surface (Fig. 4J). These results demonstrated that loss of Sema3A in vivo delayed wound healing, indicating the role of Sema3A in recovery of the epidermis.

### Enhancement of keratinocyte migration upon Rb-Sema3A treatment

**Fig. 5:**
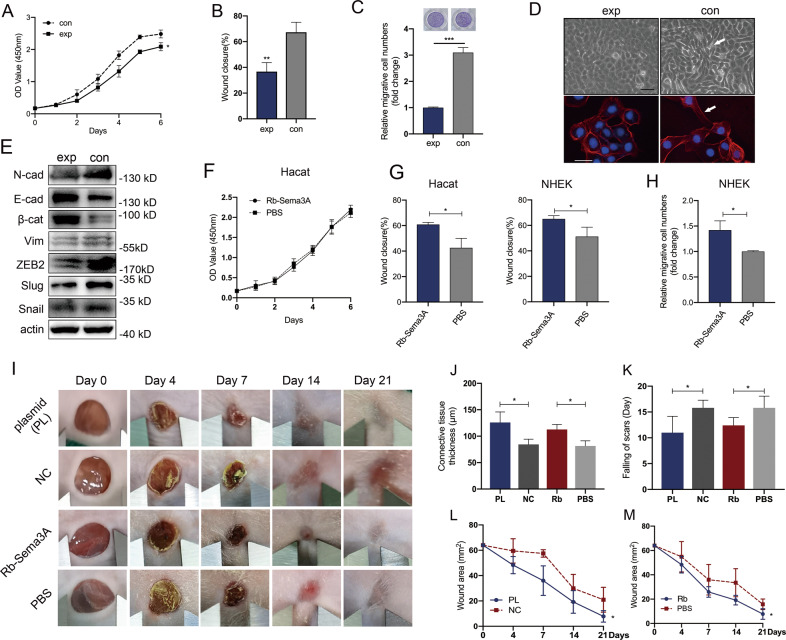
Enhancement of keratinocyte migration upon Rb-Sema3A treatment. Epithelial cells extracted from the injured (Day 4) margin of K14-Cre^TM+^;*Sema3A*^*L/L*^ and K14-Cre^TM-^;*Sema3A*^*L/L*^ mice were cultured, and the proliferation and migration capacity was determined by CCK-8 (**A**), wound healing (**B**) and Transwell assays (**C**) in vitro. **D** Morphology of keratinocytes. Immunofluorescence staining of F-actin in cells from the K14-Cre^TM+^;*Sema3A*^*L/L*^ (exp) and K14-Cre^TM-^;*Sema3A*^*L/L*^ (con) groups. White arrows point to spindle morphological alterations in the control group. **E** Western blot analysis of EMT markers by Day 4 after injury. Lysates were extracted from the injured margins of *sema3A* cKO or control mice. **F** The effect of Rb-Sema3A on the proliferation potential of Hacat cells was analysed by CCK-8. Data are shown as means ± SEM; *n* = 4. **G** Wound healing assays were performed in Rb-Sema3A-incubated Hacat and NHEK cells. The percentage of wound closure is displayed as the mean ± SEM; *n* = 3. **H** Transwell assays showed that incubated with recombinant Seam3A enhanced the migratory ability of NHEK cells. Bars indicate the mean fold changes ± SEM relative to the corresponding control; *n* = 3. **I** Two 8-mm excisional wounds were created on the back of each 7–8-week-old BALB/c nude mouse. Sema3A-transfected Hacat cells or recombinant Sema3A proteins as well as the relative control were injected subcutaneously in the margin (2 mm from the incision) of the wound in nude mice. Photographs were taken at Days 0, 4, 7, 14 and 21. The thickness of the connective tissues (**J**), time of scar falling (**K**) and area of wound are displayed (**L**, **M**). *P < 0.05; **P < 0.01; ***P < 0.001.

To further determine the role of Sema3A knockdown in the migration and invasion of keratinocytes, we isolated the epidermis of K14-Cre^TM+^;*Sema3A*^*L/L*^ mice and K14-Cre^TM-^;*Sema3A*^*L/L*^ mice and cultured these samples in vitro to compare their cell proliferation and migration capacity (Fig. 5A–C). In line with the in vivo results, keratinocytes in the experimental group grew much slower, and wound closure was significantly suppressed. We then observed the effect of Sema3A on cell morphology. Sema3A knockdown displayed compact colony formation in contrast to a well-spread and migratory phenotype in the control group (Fig. 5D). It has been well elucidated that EMT can induce the transient transition of epithelial cells to a more migratory phenotype, which is critical for rapid re-epithelialization of injured epithelium. We then tested EMT markers in cells isolated from K14-Cre^TM+^;*Sema3A*^*L/L*^ mice and K14-Cre^TM-^;*Sema3A*^*L/L*^ mice. Lower protein levels of N-cadherin, vimentin and ZEB2 were detected in the K14-Cre^TM+^;*Sema3A*^*L/L*^ group than in the control at Day 4 postinjury (Fig. 5E). In the previous immunostaining, we discovered an extremely extracellular expression of Sema3A during wound healing (Fig. 4F). We assumed that secretory Sema3A proteins could play a leading role in the process of wound healing in a non-autonomous strategy. Therefore, we introduced exogenous Sema3A recombinant protein (Rb-Sema3A) into Hacat and NHEK cells. Although Rb-Sema3A-treated cells did not show an obvious cell proliferation (Fig. 5F), the number of migrated and invasive Rb-Sema3A-treated cells was significantly higher than that of parental PBS-treated cells (Fig. 5G, H). To further confirm the function of secreted Sema3A proteins during wound healing, we injected Sema3A-transfected Hacat cells or individual recombinant Sema3A proteins subcutaneously into the margin of the wound in nude mice (Fig. 5I). Wounds in the group with Sema3A plasmid transfection closed much quicker than those in the control group. Rb-Sema3A also contributed to wound repair. The connective tissues were much thicker in the Sema3A plasmid transfection group, indicating a better healing process after injury. Consistently, subcutaneous injection of recombinant Sema3A protein significantly stimulated the process of wound healing, with thicker connective tissue and faster cutaneous repair (Fig. 5J–M).

### Successive recruitment of Sema3A and NRP1 proteins during the process of wound healing

**Fig. 6:**
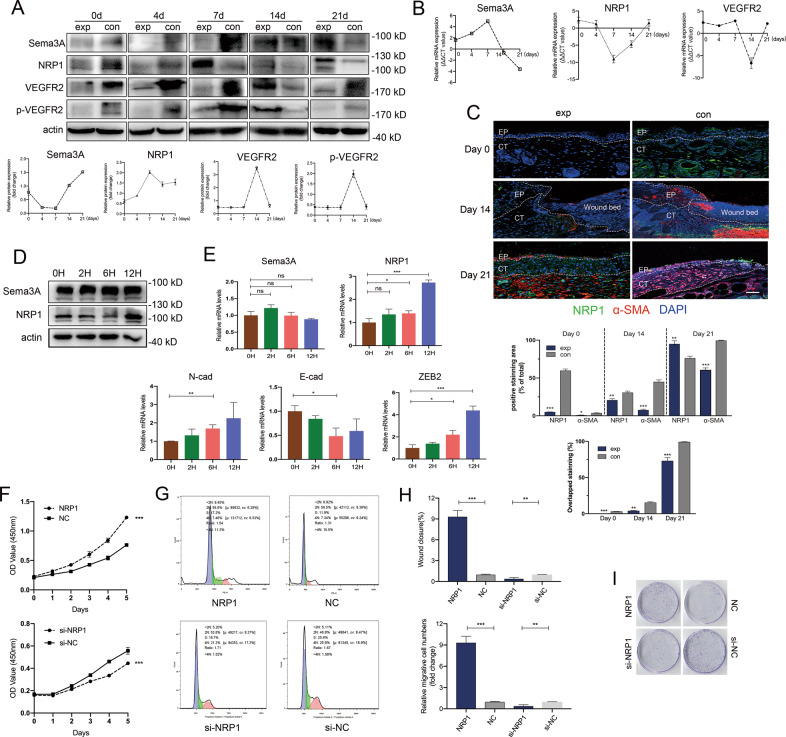
Successive recruitment of Sema3A and NRP1 proteins during the process of wound healing. **A** Schematic representation of the wound-healing studies performed in K14-Cre^TM+^;*Sema3A*^*L/L*^ and K14-Cre^TM-^;*Sema3A*^*L/L*^ mice. **B** qRT-PCR analysis of epidermal Sema3A mRNA after tamoxifen induction. GAPDH served as control. Bars indicate the mean fold changes ± SEM relative to the control (K14-Cre^TM-^;*Sema3A*^*L/L*^ group); *n* = 3. **C** Immunofluorescence of NRP1 and α-SMA at the indicated time points (left). The positive staining area (% of total) and percentage of overlapping staining (right) are demonstrated. Data are shown as means ± SEM; *n* = 3. Scale bar = 100 µm. **D** Western blot analysis of Sema3A and NRP1 after scratching at the indicated time points. **E** mRNA expression levels of Sema3A, NRP1, N-cad and E-cad after scratching. Bars indicate the mean fold changes ±  SEM relative to the control (0H). *n* = 3. **F** A CCK-8 assay was performed to assess cell growth upon NRP1 overexpression or depletion. Data are shown as means ± SEM; *n* = 5. **G** Cell cycle analysis by flow cytometry. **H** Wound healing and Transwell assays in NRP1-overexpressing or NRP1-silenced cells. **I** Colony formation experiment. *P < 0.05; **P < 0.01; ***P < 0.001.

Sema3A is an alternative ligand for NRP1. Several studies have indicated that Sema3A can bind to NRP1 and activate signal transduction mediated by NRP1-plexin interplay [33]. However, the interactions between NRP1 and its ligands and plexins in the process of wound healing are not fully understood. Here, we first tested the mRNA and protein levels of Sema3A, NRP1, VEGFR2 and p-VEGFR2 in the dermal tissues of transgenic mice at 0, 4, 7, 14 and 21 days postinjury (Figs. 6A, B and S4). On Day 0, the Sema3A, NRP1, VEGFR2 and p-VEGFR2 protein expression levels in the K14-Cre^TM+^;*Sema3A*^*L/L*^ group were much lower than those in the control group. Interestingly, we found that the protein level of Sema3A in the K14-Cre^TM+^;*Sema3A*^*L/L*^ group decreased over time and reached its lowest level by Day 7, while increased in the following period of time. However, the NRP1 protein level was strikingly increased during wound healing in Sema3A knockout mice. The levels of VEGFR2 and p-VEGFR2 showed no change (or an increase on Day 14) (Fig. 6A). The alteration of the mRNA levels of Sema3A, NRP1 and VEGFR2 was nearly consistent with the change in protein levels (Fig. 6B). The expression levels of Sema3A, NRP1 and VEGFR2 remained steady in blood and muscle tissues after wounding (Fig. S3C, D). Whereas NRP1 has been considered as a major component of the Sema3A receptor complex, we elicited the potential role of NRP1 during wound healing. K14-Cre^TM+^;*Sema3A*^*L/L*^ wounds had much lower NRP1 intensity than K14-Cre^TM-^;*Sema3A*^*L/L*^ wounds at Day 0 postinjury (Fig. 6A), and the intensity was constantly increased in the process of wound healing, along with the dermal fibroblast protein α-SMA (Fig. 6C). Thus, enrichment of NRP1 may be involved in keratinocyte migration and matrix remodeling, and its downregulation may contribute to the rate of wound healing. To confirm the effect of Sema3A and NRP1 in the early stage of wound repair, we scratched cells in a 6-well plate and tested the protein and mRNA expression levels of Sema3A, NRP1 as well as EMT-related markers. After scrubbing, the protein and mRNA levels of Sema3A were almost unchanged in a 12 h period, while NRP1 showed an enhansive expression at 12 h (Fig. 6D, E). The mRNA expression levels of N-cadherin and ZEB2 were consistent with the expression of NRP1 (Fig. 6E). These results indicated the successive recruitment of Sema3A and NRP1 proteins after injury. NRP1 instead of Sema3A played a prior role during the early stage of wound healing.

To further verify the function of NRP1 in keratinocytes, we knocked down the expression of NRP1 by RNAi in Hacat cell lines. NRP1-depleted cells exhibited a significant impairment in viability and proliferation compared with control cells (Fig. 6F, I). Flow cytometry analysis revealed a significantly decreased number of S-phase cells following NRP1 knockdown (Fig. 6G). Furthermore, NRP1 depletion diminished the migration and invasion capacity of keratinocytes (Fig. 6H). Notably, complementary to gene knockdown experiments, we found that NRP1 overexpression was sufficient to confer proliferative and migrative ability to keratinous cells and to enhance their viability.

### Interaction between NRP1 and EGFR signaling in keratinocytes

**Fig. 7:**
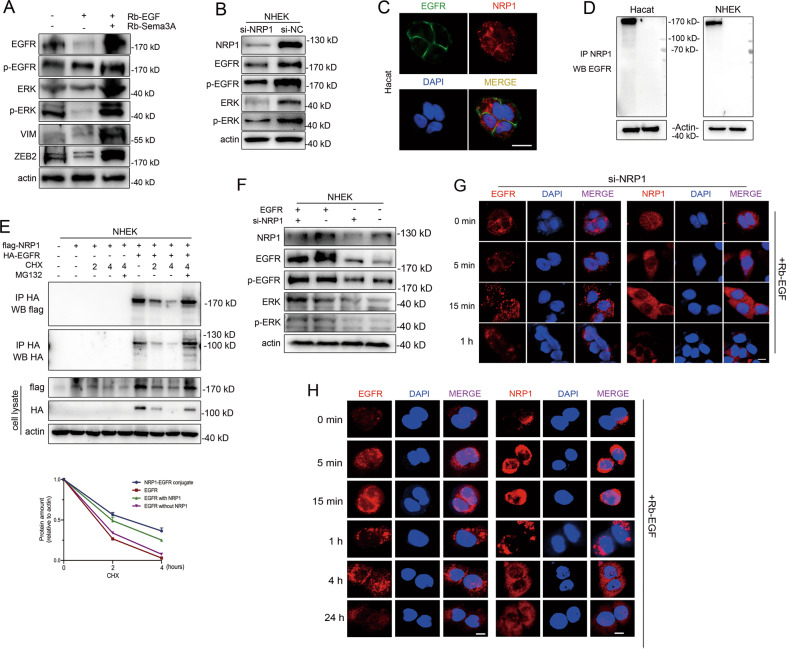
Interaction between NRP1 and EGFR signaling in keratinocytes. **A** Detection of the EGFR-ERK pathway and EMT inducers by western blotting in Hacat cells incubated with recombinant Sema3A and EGF for 48 h. **B** Protein levels of NRP1, EGFR, p-EGFR, ERK and p-ERK in cells transfected with si-NRP1. **C** Localization of NRP1 and EGFR proteins in Hacat cells. Scale bar = 20 µm. **D** Co-IP experiment between NRP1 and EGFR. IP: NRP1. WB: EGFR. **E** EGFR- and NRP1-overexpressing Hacat cells were treated with cycloheximide (CHX) for the indicated time periods to inhibit de novo protein synthesis. As a control, MG132 was added to block the catalytic activity. **F** si-NRP1 and EGFR plasmids were cotransfected into NHEK cells for 48 h. Protein levels of NRP1, EGFR, p-EGFR, ERK and p-ERK were determined by western blot. **G** NHEK cells were transfected with si-NRP1 plasmids for 2 days before EGF (50 ng/ml) stimulation. Then the IF analysis of EGFR or NRP1 was showed. Scale bar = 20 µm. **H** NHEKs were stimulated with EGF (100 ng/ml) for the indicated periods of time. IFs were subsequently conducted in the resulting cells to monitor EGFR and NRP1 localization/expression. Scale bar = 20 µm.

Recombinant human Sema3A has been reported to potentiate TGF-β1’s profibrotic effects and remodel the wounded cornea. Here, we examined the impact of Rb-Sema3A in keratinocytes with or without TGF-β1 exposure. Interestingly, after 3 days of culture, we discovered a morphological change in human recombinant Sema3A and/or TGF-β1 treatment (Fig. S5A). The number of migrated Sema3A- and/or TGF-β1-treated cells was significantly higher than that of parental PBS-treated cells (Fig. S5B, C). Meanwhile, cells grew much faster in the group treated with recombinant Sema3A and/or TGF-β1 compared with the control (Fig. S5D). However, we did not observe a significant synthetic effect with combined Rb-Sema3A and TGF-β1 treatment compared with single dose administration. We then performed qRT-PCR to assess the mRNA levels of Sema3A, NRP1, EGFR, ERK and EMT-related genes (Fig. S5E). Cells treated with Rb-Sema3A expressed higher NRP1 levels than cells treated with PBS. The mRNA level of EGFR was consistent with Sema3A expression. Western blot analysis showed that recombinant Sema3A resulted in an enhancement of ZEB2, EGFR and ERK protein expression (Fig. S5F). However, coculture with Sema3A and TGF-β1 decreased the protein expression levels of ZEB2, EGFR and ERK signaling pathway. Moreover, combined administration of recombinant Sema3A and EGF generated protein levels of ZEB2, p-EGFR and p-ERK that were much higher than those achieved by incubation with Sema3A or EGF alone (Fig. 7A). As NRP1 is a coreceptor for multiple extracellular ligands, we further explored the potential binding ligands, except for Sema3A. In cancer cells, the extracellular domain of NRP1 can interact with EGFR and promote the EGFR signaling cascade elicited upon EGF stimulation [34]. To determine whether NRP1 is in complex with EGFR in keratinocytes, we transfected NRP1 siRNA into Hacat and NHEK cells. Notably, we observed a depletion of EGFR and ERK protein expression as well as their phosphorylation products due to NRP1 silencing (Fig. 7B, G). To discover the interaction between NRP1 and EGFR in keratinocytes, we first examined the colocalization of NRP1 and EGFR by confocal microscopy. Confocal fluorescence microscopy analysis in nonpermeabilized conditions revealed the presence of large EGFR clusters on the cell surface (Fig. 7C). NRP1 was mainly present on the cell surface and rarely dispersed in the cytoplasm (Fig. 7C). Co-immunoprecipitation experiments indicated that NRP1 interacted with EGFR upon NRP1 overexpression (Fig. 7D). EGFR- and NRP1-overexpressing Hacat cells were treated with cycloheximide (CHX) for the indicated time periods to inhibit de novo protein synthesis. As a control, MG132 was added to block the catalytic activity. EGFR was almost completely degraded within 4 h, and upon inhibition of the proteasome, a recovery of EGFR protein levels was detected. The EGFR-NRP1 conjugate was degraded more slowly (Fig. 7E). We then transfected full-length EGFR plasmids into NRP1-depleted cells and examined the EGFR-ERK signal. Immunoblotting results demonstrated that the upregulation of EGFR increased the protein levels of EGFR, whereas NRP1 depletion restrained the upregulation of EGFR-ERK activity induced by EGFR (Fig. 7F). To assess the positive regulation between NRP1 and EGFR in the presence of EGF, we performed immunofluorescence in NHEK cells. Confocal microscopy showed that EGF stimulated the activation of both EGFR and NRP1 (15 min) with a significant increase in nuclear localization and gradual degradation in lysosomes (Fig. 7H). Furthermore, we added the ERK inhibitor U0126 to NHEKs for 2 days. Immunostaining of EGFR (Fig. S6A) showed a significant increase in EGFR-positive peripheral puncta (without nuclear activation) following EGF incubation. Immunostaining analyses of NRP1 were consistent with EGFR staining, with a decrease in active nuclear orientation (Fig. S6A). Simultaneous U0126 and EGF treatment failed to activate NRP1 in NHEK cells (Fig. S6B). These results indicated that Rb-Sema3A could work synergistically with EGF to co-activate NRP1 and EGFR.

### Synergetic effect of Rb-EGF and Rb-Sema3A through EGFR/ERK signaling regulated by NRP1

**Fig. 8:**
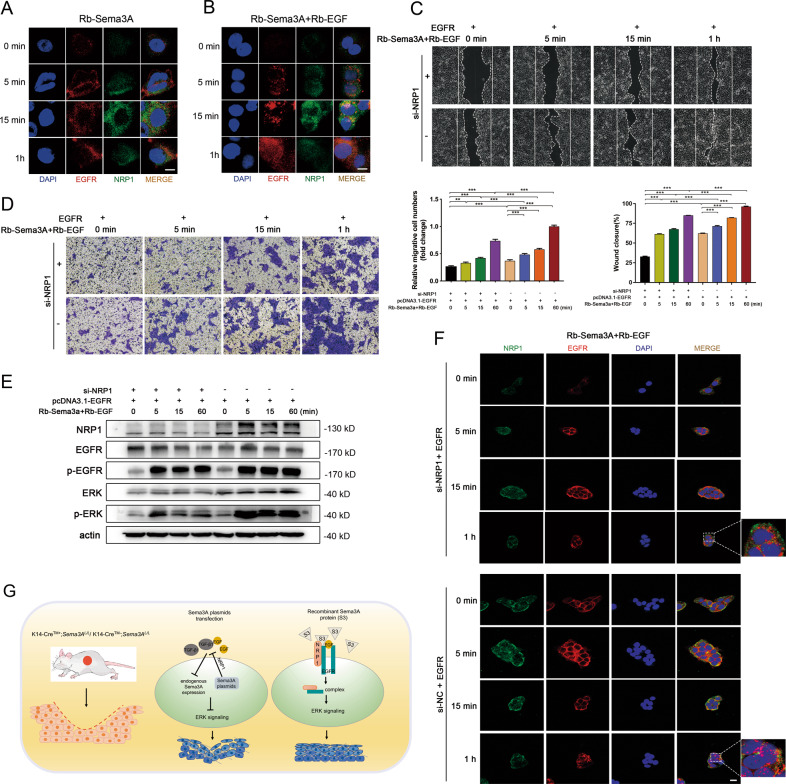
Synergetic effect of Rb-EGF and Rb-Sema3A through EGFR/ERK signaling regulated by NRP1. **A** NHEKs were serum starved and subsequently stimulated with Rb-Sema3A for 5 min to 1 h and subjected to IF analyses. **B** Combined treatment with Rb-EGF and Rb-Sema3A was utilized in keratinocytes at the indicated time points. EGFR and NRP1 localization/expression was shown by IF staining. Wound healing assays (**C**) and Transwell assays (**D**) were performed in Rb-Sema3A-,Rb-EGF and EGFR plasmids incubated in Hacat cells after transfected with si-NRP1 or negative control. The percentage of wound closure is displayed as the mean ± SEM; *n* = 3. Bars indicate the mean fold changes ± SEM relative to the corresponding control; *n* = 3. **E** Western blot analysis of EGFR-ERK pathway after treatment at the indicated time points. **F** Immunofluorescence in Hacat cells. si-NRP1 or si-NC plasmids were transfected in EGFR-overexpressing cells. Recombinant EGF and Sema3A cytokines were added in cells to test the process of NRP1 and EGFR activation and degradation. Scale bar = 20 µm. **G** A schematic of the proposed mechanism. **P < 0.01; ***P < 0.001.

To further determine whether Rb-Sema3A proteins promotes migration of keratinocytes through the interaction between NRP1 and EGFR, intensity of NRP1 and EGFR were detected by immunofluorescence. Mutual localization was detected in Fig. 8A after Rb-Sema3A treatment and the co-treatment of Rb-EGF and Rb-Sema3A intensified this effect (Fig. 8B). In addition, si-NRP1 or negative control plasmids were introduced in the EGFR transfected cells, then the cells were treated with Rb-EGF and Rb-Sema3A with indicated times. Cell migration capacity was determined in Fig. 8C, D. Results showed that si-NRP1 delayed the cell migration rate. Western blot analysis confirmed this morphological alteration (Fig. 8E). Moreover, we performed immunofluorescence in Hacat cells. si-NRP1 limited the activation of EGFR cascades in the cytoplasm and accelerated the degradation of EGFR (Fig. 8F). Overall, these results demonstrated that Rb-EGF together with Rb-Sema3A resulted in the activation of NRP1 and EGFR cascades, thus promoting cell migration ability. In summary, these findings indicated that the combination of Sema3A with EGF proteins could maintain the activation of EGFR by interacting with NRP1 and thereby regulate the internalization of the EGFR-NRP1 complex.

## Discussion

Cutaneous wounding presents a unique challenge whereby the epidermis must alter its proliferative, migratory, and differentiating dynamics to re-establish a functional permeability barrier [35]. Directional migration of epithelial cells is a crucial component of tissue regeneration and repair [36]. The re-epithelialization process is initiated with a wedge-shaped mass of keratinocytes that move across the granulation tissue and continues until keratinocytes from opposing sides of the wound reestablish contact [37]. As a family of secreted proteins, several hypotheses have proposed how Sema3A might be recruited to and retained in the process of cell movements, including the inhibition of cortical neurons and growth cone collapse, thus providing chemorepulsive guidance for migrating axons [16]. Repeated application of Sema3A ointment significantly inhibited scratching behavior and improved dermatitis scores in mice, thus improving the pathophysiology of atopic dermatitis (AD) [25]. However, the function of Sema3A in skin regeneration and functional recovery after wound healing has not been well elucidated.

Because Sema3A plays a complex role in the body, we managed to mimic the two potential ways concerning Sema3A overexpressoin and clarified the mechanism on the two strategies. In cancer cells, full-length Sema3A plasmids transfection suppressed tumorigenesis and blocked metastasis of tumor cells that were implanted into mice. Sema3A has also been shown to inhibit the migration of breast cancer cells and invasiveness of prostate cancer cells in vitro [38]. In our study, we demonstrated that Sema3A adenovirus plasmids transfection limited keratinocyte proliferation and decreased migrative capacity, as assessed by an in vitro wound healing assay. Inhibition of Sema3A by RNAi significantly promoted cell growth and migration. NRP1 was originally identified as a receptor for class-3 semaphorins controlling neuronal guidance and axonal growth. In addition, NRP1 is expressed in keratinocytes and guides cell proliferation and movement. Mice lacking NRP1 in the epidermis are more sensitive to UVB irradiation and display increased apoptosis following irradiation [39]. In cancer cells, NRP1 overexpression was associated with poor prognosis and lymph node metastasis [40]. In our study, results revealed that NRP1 played a positive role during keratinocyte proliferation and wound healing in vivo and in vitro. To explore the level of NRP1 in response to Sema3A transfection, we introduced Sema3A-overexpressing adenovirus or RNAi into keratinocytes and determined the expression level of NRP1. Blockade of NRP1 reversed Sema3A-restrained cell migration, which indicated that the NRP1-mediated pathway was essential in cell movement. We further showed that the NRP1-dependent regulation of Sema3A led to confinement of the EGFR/ERK pathway. Utilizing selective inhibition, U0126, we observed that enhancement of cell migration in Sema3A-deficient keratinocytes is dependent on EGFR/ERK signaling.

As a secretive protein, recombinant Sema3A usually require one or more receptors and co-receptors to achieve biological processes. In this study, we utilized mice with K14-Cre-mediated deletion of Sema3A and investigated the alteration of proliferation and migration during wound healing. Interestingly, Sema3A depletion caused postponement of wound closure with decreased re-epithelialization and matrix growth. After injury, the expression level of Sema3A increased in the first 7 days and then decreased during healing in the control and wildtype groups. Sema3A is a secreted protein and participates in various physiological processes. Upon knockdown, the endogenous Sema3A-NRP1/Plexin pathway expression almost undetectable in the epidermis. After wound occurred, Sema3A proteins from epidermis in the control group can be secreted from keratinocytes and promoted keratinocytes migration and wound repair. By contrast, Sema3A depletion limited the wound repair after injury. To further determine the non-autonomous effect of Sema3A during cutaneous wound healing, we injected Hacat cells expressing Sema3A plasmids or recombinant Sema3A cytokines in nude mice and detected the wound repair rate after punching. Contrary to the result obtained from Sema3A transfection, in vivo experiments suggested a positive wound healing effect of secreted Sema3A during wound healing.

Previous studies showed that the application of Sema3A significantly decreased the numbers of infiltrating eosinophils and CD4 + T cells and tended to decrease the number of mast cells. In the dorsal wound bed of K14-Cre^TM+^;*Sema3A*^*L/L*^ mice, we discovered severe infiltration and immunoreactive vessels representing endothelial cell adhesion molecules. During pathological conditions such as wound repair and/or inflammation, Sema3A may suppress VEGF-mediated angiogenesis while enhancing vascular permeability [21]. Sema3A and VEGF share a common coreceptor, NRP1, providing competing binding between the two ligands. In vivo experiments showed that decreases in VEGFR2 and p-VEGFR2 resulted in abnormal microvessel outgrowth and vascular permeability.

It has long been recognized that epithelial cells possess a range of inherent plasticity, including the ability to become mesenchymal cells. Generally, EMT-related studies have examined the expression of epithelial (i.e., E-cadherin) and mesenchymal (i.e., N-cadherin, vimentin, Snail, Slug and ZEB2) markers to define this process [11]. Growth factors and signaling cascades induce EMT in a tissue- and context-dependent manner, including TGF-β, EGF and fibroblast growth factor (FGF). Sema3A regulated the expression of cell-cell junctional molecules in keratinocytes and corneal epithelial cells [24]. Interestingly, although recombinant Sema3A protein administration caused a spindle-shaped alteration in keratinocytes, EMT markers (including Snail and Vimentin) showed lower expression levels than the control. We then sought a potential factor that coordinated Sema3A in the promotion of cell migration. By performing qRT-PCR and western blot, we accidentally discovered an enhancement of EGFR expression. Combined treatment with recombinant Sema3A and EGF greatly activated the EGFR signaling pathway and EMT inducers (vimentin and ZEB2, Fig. 7A). It has been reported that Sema3A transiently triggers EGFR phosphorylation in a Plexin A2-dependent manner [41]. Given the role of NRP1 as a multifunctional coreceptor with the ability to bind disparate ligand families, this has sparked new areas of research implicating NRPs in diverse biological functions. Apart from class 3 semaphorins and VEGF, NRP1 co-interacts with other important heparin-binding cytokines, such as EGF and EGFR, hepatocyte growth factor (HGF) and its receptor c-Met [18]. In cancer cells, NRP1 mediates ligand-induced EGFR clustering and endocytosis, leading to intracellular activation of the AKT signaling cascade [34]. Combined recombinant EGF and Sema3A treatment significantly activated EGFR/ERK pathway as well as EMT regulators (Fig. 7A). These results demonstrated the synergetic effect of EGF and Sema3A co-treatment may accelerate wound healing upon cutaneous injury. To demonstrate whether recombinant Sema3A protein promoted migration of keratinocytes through the interaction between NRP1 and EGFR, full-length EGFR plasmids were transfected into NRP1-depleted Hacat cells upon simultaneous stimulation of Rb-Sema3A and Rb-EGF. The migration capacities of Hacat cells induced by stimulation in time-dependent manner were restrained by NRP1 depletion. Meanwhile, lower levels of p-ERK were observed (Fig. 7F). These above results demonstrated that exogenously applied EGF together with Sema3A resulted in the activation of NRP1 and EGFR cascades, thus promoting cell migration ability.

In conclusion, these results indicated a paradoxical role of autonomous and non-autonomous Sema3A during wound healing (Fig. 8G). Our study indicated a new mechanism considering dual NRP1-EGFR signal activation in which combined administration of recombinant EGF and Sema3A protein could accelerate wound closure effectively, thus providing promising treatment prospects in the future.

## Materials and methods

### Mice

Mice expressing floxed Sema3A were purchased from RIKEN BRC (No. RBRC01106). Transgenic mice expressing the K14-cre recombinase (purchased from Shanghai Model Organisms Center, Inc.) were mated with floxed mice to obtain conditional knockout mice. To activate K14-cre, 83.5 mg/kg tamoxifen (Sigma) in corn oil (Sigma) was intraperitoneally injected once a day for 10 consecutive days to generate K14-Cre^TM+^;*Sema3A*^*L/L*^ mice. Mice without tamoxifen intervention are presented as controls. In all studies involving animals, we used both male and female mice (12 wk of age) for our experiments. We maintained all the mice under a 12 h light–dark cycle. Sample sizes were based in standard protocols in the field. Experiments were blinded to the person performing marker analysis.All animal studies were approved by the Institutional Animal Care and Use Committee at Nanjing Medical University.

### Wounding protocol

K14-Cre^TM+^;*Sema3A*^*L/L*^ and K14-Cre^TM-^;*Sema3A*^*L/L*^ mice were anaesthetized by intraperitoneal injection of 200 mL of chloral hydrate. The hair of the back of the mouse was shaved with an electric clipper followed by a depilatory cream. To make an excisional wound, the skin was rinsed with alcohol, and two excisional wounds of 8.0 mm were created in the scalp at the midline with a sterile dermal punch. After wounding, the mice were caged individually, and the wounds were not dressed. The wound area was calculated by measuring the wound size each day and is shown as a percentage of the 0-day control. Wounds were photographed, and animals were euthanized to collect the wounded tissue at the indicated time points. In all in vivo experiments, 3–6 mice per group were examined at each time point, and the value per animal was the unit of measurement.

For the nude mice, two 8-mm excisional wounds were created on the back of each 7–8-week-old BALB/c mouse and photographed at the indicated time points. Sema3A-transfected Hacat cells or individual recombinant Sema3A protein and the relative control (empty vector-transfected Hacat cells or PBS) were injected subcutaneously into the margin of the wound in nude mice. The wound area was calculated by measuring the wound size each day and is shown as a percentage of the 0-day control. Photographs were taken at Days 0, 7, 14 and 21.

### Cell culture

Hacat cells (ATCC 12191) and NHEKs (purchased from BeNa Culture Collection) at passages 2–4 were cultured in DMEM-F12 (Gibco-BRL, Grand Island, NY, USA) supplemented with 10% FBS (Gibco) at 37 °C with 5% CO2. All cell lines were regularly tested for mycoplasma contamination using the Mycoalert Mycoplasma Detection Kit (Lonza). Cells were grown to 70–80% confluence and then stimulated with recombinant human Sema3A, TGF-β1 or EGF (all purchased from Peprotech) for 48 h to determine changes in EMT proteins. Hacat and NHEK cells were stimulated with TGF-β1 or EGF in the presence or absence of erlotinib (Seleckchem) or U0126 (Seleckchem), both of which were utilized at a concentration of 10 μM, as reported in previous studies. Mouse skin keratinocytes were isolated from K14-Cre^TM+^;*Sema3A*^*L/L*^ or K14-Cre^TM-^;*Sema3A*^*L/L*^ mice cultured in DMEM-F12 medium supplemented with 10% FBS, 0.5 μg/mL hydrocortisone, 5 μg/mL insulin, 10 ng/mL EGF, 100 U/mL sodium pyruvate, 100 μg/mL penicillin/streptomycin in 5% CO2 and 32 °C. Cells between passages 2–6 were used for the experiments.

### Plasmid construction and transfection

The full-length coding region of human NRP1 or EGFR cDNA was inserted into pcDNA 3.1 vectors with a 3×FLAG-tag or HA-tag and was constructed by GenePharma (Shanghai, China). Adenovirus or plasmid vectors encoding Sema3A were generated by GenePharma. Nontargeting scramble, Sema3A siRNA and NRP1 siRNA were purchased from Santa Cruz Biotechnology. Cells utilized for transfection (5 × 10^5^ cells/well) were grown to ~60% confluence in the recommended growth medium using Lipofectamine 2000 (Invitrogen) according to the manufacturer’s protocol. The medium was replaced with preequilibrated and prewarmed fresh culture medium 4–6 h after transfection. These cells were incubated for 24–48 h before a series of experiments.

### Wound healing and transwell migration assay

Mitomycin C (10 μg/mL) was included in the medium to prevent cell proliferation. For in vitro wounds, confluent cells were scratched using a 200 μl pipette tip, and the wells were washed with PBS to remove cell debris. Photographs were taken as indicated to assess the number of keratinocytes that had migrated into the wounded area. The cell migration rate was determined by measuring the reduction in the cell-free area using ImageJ software. For the migration assay, 2 × 10^5^ cells were incubated in the upper chamber of a transwell (Corning Costar) containing a polycarbonate membrane filter (8-μm pore size). After 24 h, cells remaining on the upper surface of the membrane were removed, and migrated cells on the lower surface were fixed and stained with methylene blue. Migrating cells were quantified by counting cells in each well in three random fields (×100). Experiments were performed at least three times.

### Cell proliferation assay and colony formation assay

For the CCK-8 assay, 1 × 10^3^ cells were plated in 96-well plates. Cell viabilities were determined at the indicated time points. At the end of each timing, 10 μL of CCK-8 reagent (Dojindo, Japan) was introduced to each well and incubated for 2 h at 37 °C. Cell growth curves were plotted according to the average absorption values of each experiment. For colony formation assays, cells were plated in 6-well plates at a concentration of 300 cells/well and incubated for 2 weeks to allow colony formation. After incubation, cells were fixed with formalin and stained with crystal violet solution to visualize plaques. Cell colony formation numbers were counted under a microscope (DM4000B) and analysed by ImageJ software.

### Flow cytometry analysis

Hacat cells (1 × 10^6^ cells/well) were plated in 6-well plates. Cells were then harvested and washed twice with PBS and resuspended in 70% ice-cold ethanol for 2 days. Then, the cells were washed and centrifuged and resuspended in 0.5 mL of propidium iodide (PI) staining buffer for 30 min in the dark at room temperature. The cell cycle profiles were assessed by FACS cytometry at 488 nm.

### Western blotting

Whole-cell lysates were prepared using radioimmunoprecipitation assay (RIPA) lysis buffer containing a protease/phosphatase inhibitor cocktail. Protein concentrations in the lysates were determined using BCA reagent. The proteins in lysates or secreted into the culture medium were separated on a 10% SDS-polyacrylamide gel electrophoresis (SDS–PAGE) gel and transferred onto a polyvinylidene fluoride membrane. The membrane was incubated in 5% skim milk in Tris-buffered saline containing 0.1% Tween 20 to block nonspecific binding. The blot was incubated with appropriate primary antibodies against Sema3A (ab23393, Abcam), NRP1 (ab81321, Abcam), EGFR (#3777, CST), p-EGFR (#4267, CST), ERK (#4695, CST), p-ERK (#4370, CST), p-Smad2 (ab18338, Abcam), E-cad (#3195, CST), N-cad (ab18203, Abcam), Vimentin (#5741, CST), ZEB2 (#14026, ProteinTech), Snail (#3879, CST), Slug (#9585, CST), VEGFR2 (#9698, CST) or p-VEGFR2 (#2478, CST) for 12 h at 4 °C. The immunoreactive protein bands were incubated with HRP-conjugated anti-mouse or rabbit antibody for 1 h at room temperature and detected with ECL reagents. All the original data for WB have been demonstrated in the supplementary materials. The bands obtained were densitometrically quantified using ImageJ software.

### Immunofluorescent staining

Paraffin-embedded wound tissues were collected and sectioned perpendicular to the wound. For immunostaining of paraffin-embedded skin samples, the samples were deparaffinized and rehydrated. Keratinocytes were cultured on dishes overnight and then fixed with 4% formaldehyde in 0.1 M phosphate buffer. The antibody against Sema3A was from Abcam (ab23393); the antibody against ZEB2 was from ProteinTech (#14026); the antibody against α-SMA was from Abcam (ab7817); the antibody against NRP1 was from Abcam (ab81321) with a dilution of 1:100 at 4 °C overnight. Then, tissues or cells were washed and further incubated with FITC- or Cy3-labeled goat anti-rabbit or anti-mouse IgG (Proteintech, China) at a dilution of 1:500 at room temperature for 30 min and stained with 4′,6‐di‐amidino‐2‐phenylindole (DAPI; Sigma Chemicals). Plates or tissues were blindly examined and imaged by a fluorescence microscope (DM4000B, Leica, Germany).

### Quantitative RT–PCR

Cells and tissue samples were collected to extract total RNA using TRIzol (Invitrogen, Carlsbad, CA, USA) reagent, and cDNA was generated using Superscript (Vazyme, Nanjing, China) according to the manufacturer’s instructions. Relative expression levels of related genes were measured by the 2^−ΔΔCT^ method. Promotor sequence of EGFR was downloaded from NCBI and the potential binding transcriptional factors were predicted by Jasper(https://jaspar.genereg.net/). Primers were as follows: Sema3A: F: 5′- GTGCCAAGGCTGAAATTATCCT-3′ R: 5′- CCCACTTGCATTCATCTCTTCT-3′; NRP1: F: 5′- GGCGCTTTTCGCAACGATAAA-3′ R: 5′- TCGCATTTTTCACTTGGGTGAT-3′; VEGFR2: F: 5′- GGCCCAATAATCAGAGTGGCA-3′ R: 5′- CCAGTGTCATTTCCGATCACTTT-3′; EGFR: F: 5′- ATGAGGACATAACCAGCCACC-3′ R: 5′- AGGCACGAGTAACAAGCTCAC -3′; TCF4: F: 5′-AGAAACGAATCAAAACAGCTCCT-3′ R: 5′-CGGGATTTGTCTCGGAAACTT-3′; CEBPA: F: 5′-GTGGACAAGAACAGCAACGA-3′ R: 5′-GGTCATTGTCACTGGTCAGC-3′; CEBPB: F: 5′-CTTCAGCCCGTACCTGGAG -3′ R: 5′-GGAGAGGAAGTCGTGGTGC-3′; TP53:F: 5′- CAGCACATGACGGAGGTTGT-3′ R: 5′-TCATCCAAATACTCCACACGC-3′; XBP1:F: 5′-CCCTCCAGAACATCTCCCCAT-3′ R: 5′-ACATGACTGGGTCCAAGTTGT-3′; GATA-1: F: 5′-TTGTCAGTAAACGGGCAGGTA-3′ R: 5′-CTTGCGGTTTCGAGTCTGAAT-3′; E-cad: F: 5′-CGAGAGCTACACGTTCACGG-3′ R: 5′-GGGTGTCGAGGGAAAAATAGG-3′; N-cad: F: 5′-TCAGGCGTCTGTAGAGGCTT-3′ R: 5′-ATGCACATCCTTCGATAAGACTG-3′; ZEB2: F: 5′-CAAGAGGCGCAAACAAGCC-3′ R: 5′-GGTTGGCAATACCGTCATCC-3′; ERK: F: 5′-CTACACGCAGTTGCAGTACAT-3′ R: 5′-CAGCAGGATCTGGATCTCCC-3′; AKT: F: 5′-AGCGACGTGGCTATTGTGAAG-3′ R: 5′-GCCATCATTCTTGAGGAGGAAGT-3′; Snail: F: 5′-ACTGCAACAAGGAATACCTCAG-3′ R: 5′-GCACTGGTACTTCTTGACATCTG-3′; Slug: F: 5′-CGAACTGGACACACATACAGTG-3′ R: 5′-CTGAGGATCTCTGGTTGTGGT-3′; Vimentin: F: 5′-GACGCCATCAACACCGAGTT-3′ R: 5′-CTTTGTCGTTGGTTAGCTGGT-3′.

### Co-immunoprecipitation assay

Hacat cells were harvested and lysed in 600 μL of RIPA buffer (Beyotime) with protease inhibitors. Then, the cells were scraped on ice, and the supernatants were collected by centrifugation. The supernatants of cell lysates were incubated with the indicated antibodies, EGFR (#3777, CST), NRP1 (ab81321, Abcam), Flag (ab205606, Abcam), or HA (ab9110, Abcam), and Protein A/G PLUS-Agarose beads (Sigma-Aldrich) at 4 °C for 12 h. After extensive washing, immunoprecipitated samples were subjected to SDS–PAGE and western blotting.

### Statistical analysis

For cell experiments, none of the samples was excluded. For animal experiments, none of the animals was excluded from the analysis except for animals that were dead or no enough sample were collected. In vitro experiments were repeated a minimum of 3 times in triplicate and performed by GraphPad Prism 8. Significant differences between the experimental and control groups were determined using unpaired t-tests. Statistical tests, chosen based on the nature of the comparison being made and the standard tests used in the field, are indicated in the figure legends. Underlying assumptions for these tests, including sample independence, variance equality, and normality were assumed to be met although not explicitly examined. All measurements were taken from distinct samples, as noted in figure legends, and no data were excluded. Sample sizes were based in standard protocols in the field. Unless otherwise stated, at least three biological independent replicates were performed for each experiment. Asterisks denote *p* value as follows: *p < 0.05, **p < 0.01, ***p < 0.001, ****p < 0.0001.

## Supplementary information

aj-checklist

Supplementary material-figure legends

Figure S1

Figure S2

Figure S3

Figure S4

Figure S5

Figure S6

Original Data for WB

## Data Availability

The datasets used and/or analyzed during the current study are available from the corresponding author on reasonable request.
