# Supplementary material for: Regulation of Semaphorin3A in the process of cutaneous wound healing
Source: Cell Death Differ. 2022 Mar 26;29(10):1941–54. doi: 10.1038/s41418-022-00981-6 (PMC9525670; doi:10.1038/s41418-022-00981-6)
Supplement: Supplementary file 2 — Supplementary material-figure legends [file 41418_2022_981_MOESM2_ESM.docx]

**Supplementary figure legends**

**Fig. S1.** Verification of the knock-out of K14-Cre;*Sema3A^L/L^* mice. Asterisk (*) represents the identification of loxp/loxp or Cre transgenic mice.

**Fig. S2. (A)** EGF accelerated cell proliferation in a dose-dependent manner. Data are shown as means ± SEM; n = 3. **(B)** Western blot results showed that EGFR signalling was activated along with EMT inducers. **(C)** A schematic diagram of the predicted binding sites of transcriptional factors to the EGFR promoter sequences.

**Fig. S3. (A-B)** Relative Sema3A mRNA expression levels in blood **(A)** and muscular **(B)** tissues in K14-Cre^TM+^;*Sema3A^L/L^* and K14-Cre^TM-^;*Sema3A^L/L^* mice. Bars indicate the mean fold changes ± SEM relative to control (K14-Cre^TM-^;*Sema3A^L/L^* group); n=3. Percentage of wound closure is displayed by means ± SEM; n = 3. **(C-D)** Relative levels of Sema3A mRNA in the blood **(C)** or muscle **(D)** in indicated time points after injury.

**Fig. S4.** Expression of Sema3A, NRP1, VEGFR and p-VEGFR in K14-Cre^TM-^;Sema3A^L/L^, K14-Cre^TM+^;Sema3A^L/L^ and wild-type mice as determined by Western blotting.

**Fig. S5. (A)** Morphological alteration with human recombinant Sema3A and/or TGF-β1 treatment. **(B-C)** Wound healing assay **(B)** and Transwell assay **(C)** in cells treated with exogenous Sema3A and/or TGF-β1. The percentage of wound closure is shown as the mean ± SEM; n = 3. **(D)** A CCK-8 assay was performed to assess cell growth during incubation with Sema3A and/or TGF-β1. Data are shown as means ± SEM; n = 3. **(E)** Cells were incubated with recombinant Sema3A and/or TGF-β for 48 hr, and the mRNA levels of NRP1, Sema3A, Snail, E-cad, ERK, AKT, N-cad, ZEB2, vimentin, Slug and EGFR were determined. Bars indicate the fold changes ± SEM relative to the control (PBS group). **(F)** Protein levels of NRP1, EMT-related genes and the EGFR signalling pathway were demonstrated following treatment with Sema3A and/or TGF-β1. *P < 0.05; **P < 0.01; ***P < 0.001.

**Fig. S6 (A)** U0126 (10µM) was utilized for 2 days in NHEK cells before EGF (50ng/ml) stimulation. And the IF analysis of EGFR or NRP1 was showed. **(B)** U0126 (10µM) and EGF (50ng/ml) was simultaneously applied in NHEK cells, and EGFR or NRP1 staining was demonstrated. Scale bar:20µm.
