# Supplementary figures and images for: Regulation of Semaphorin3A in the process of cutaneous wound healing
Source: Cell Death Differ. 2022 Mar 26;29(10):1941–54. doi: 10.1038/s41418-022-00981-6 (PMC9525670; doi:10.1038/s41418-022-00981-6)

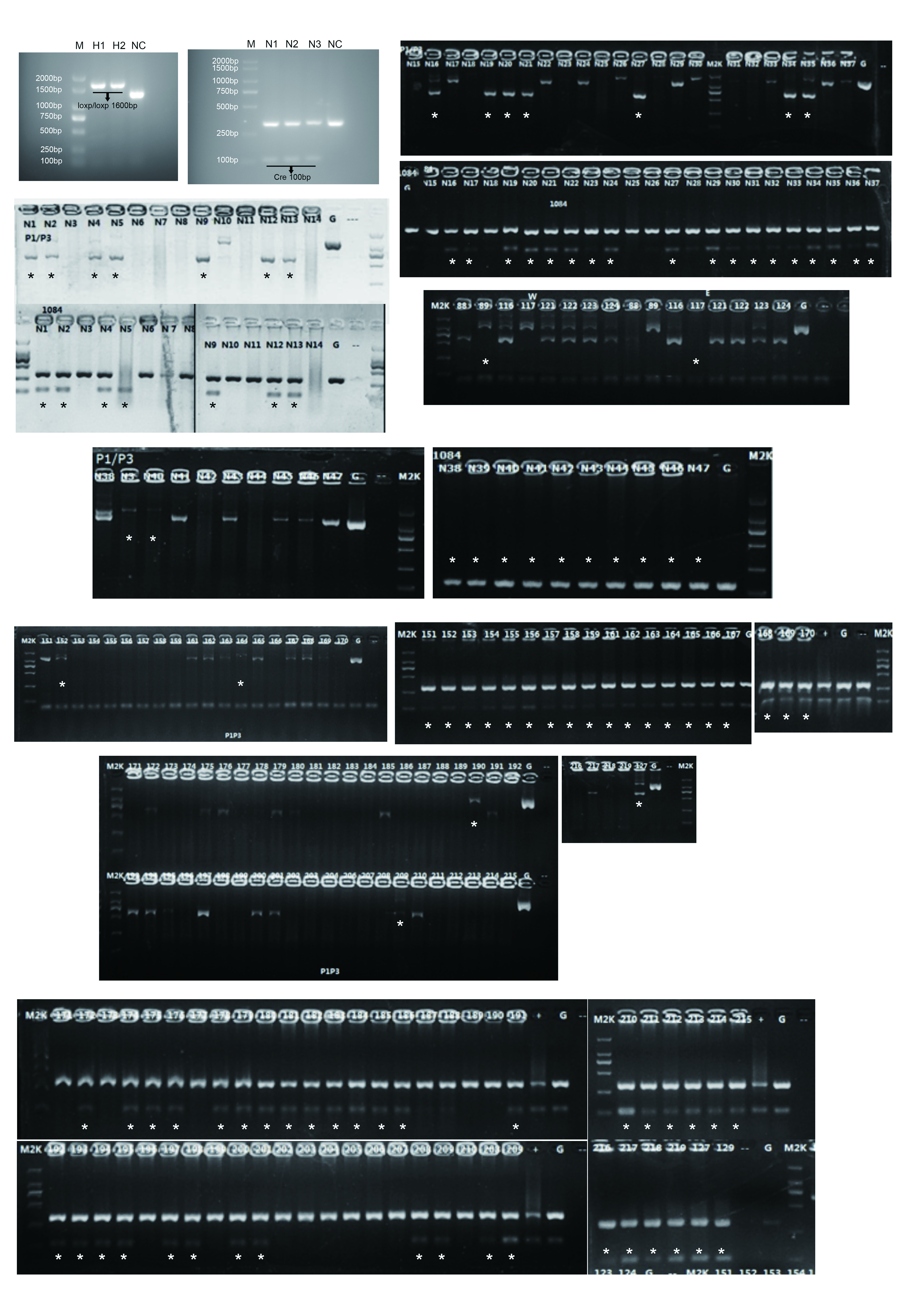

Supplement: Supplementary file 3 — Figure S1 [file 41418_2022_981_MOESM3_ESM.tif]

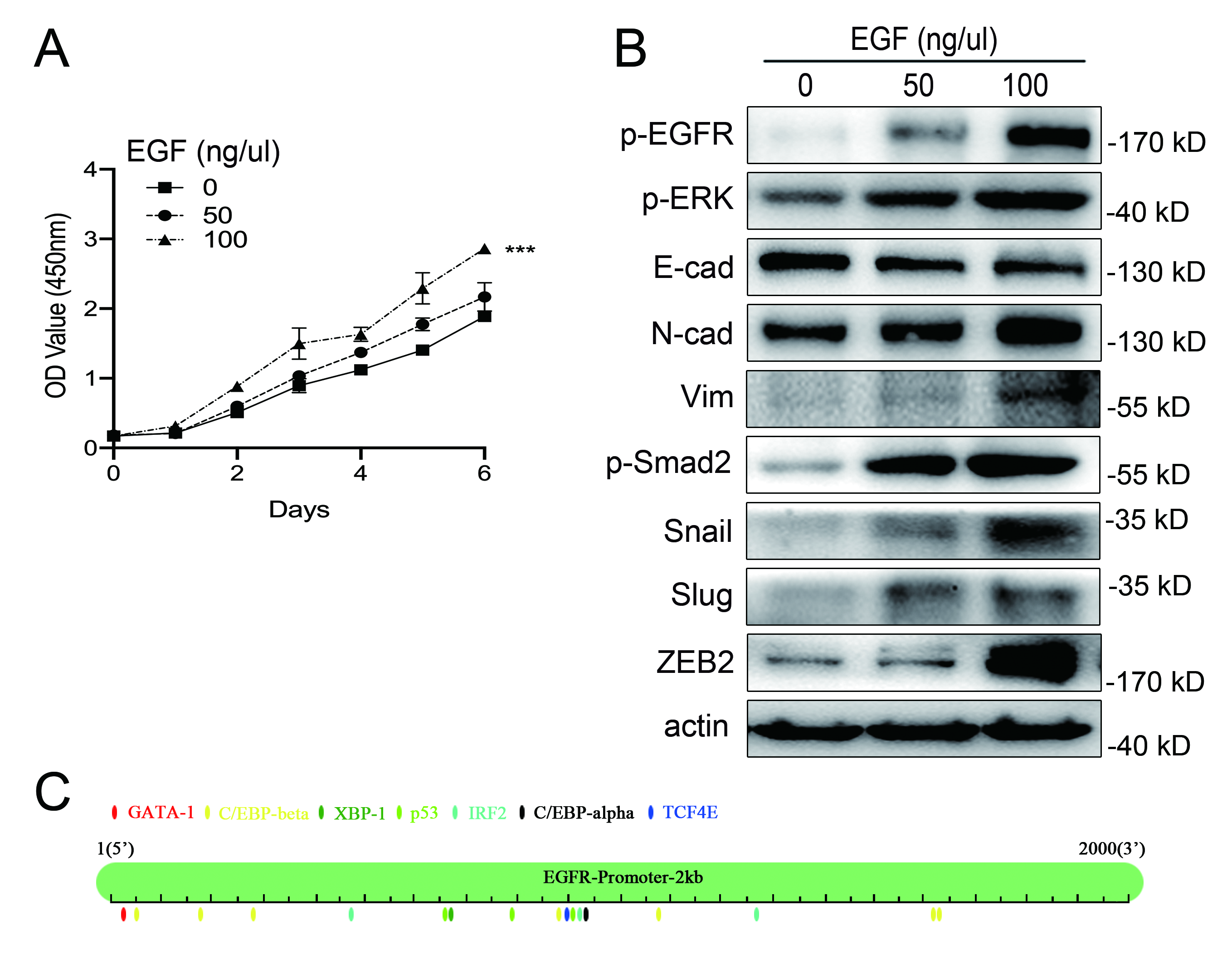

Supplement: Supplementary file 4 — Figure S2 [file 41418_2022_981_MOESM4_ESM.tif]

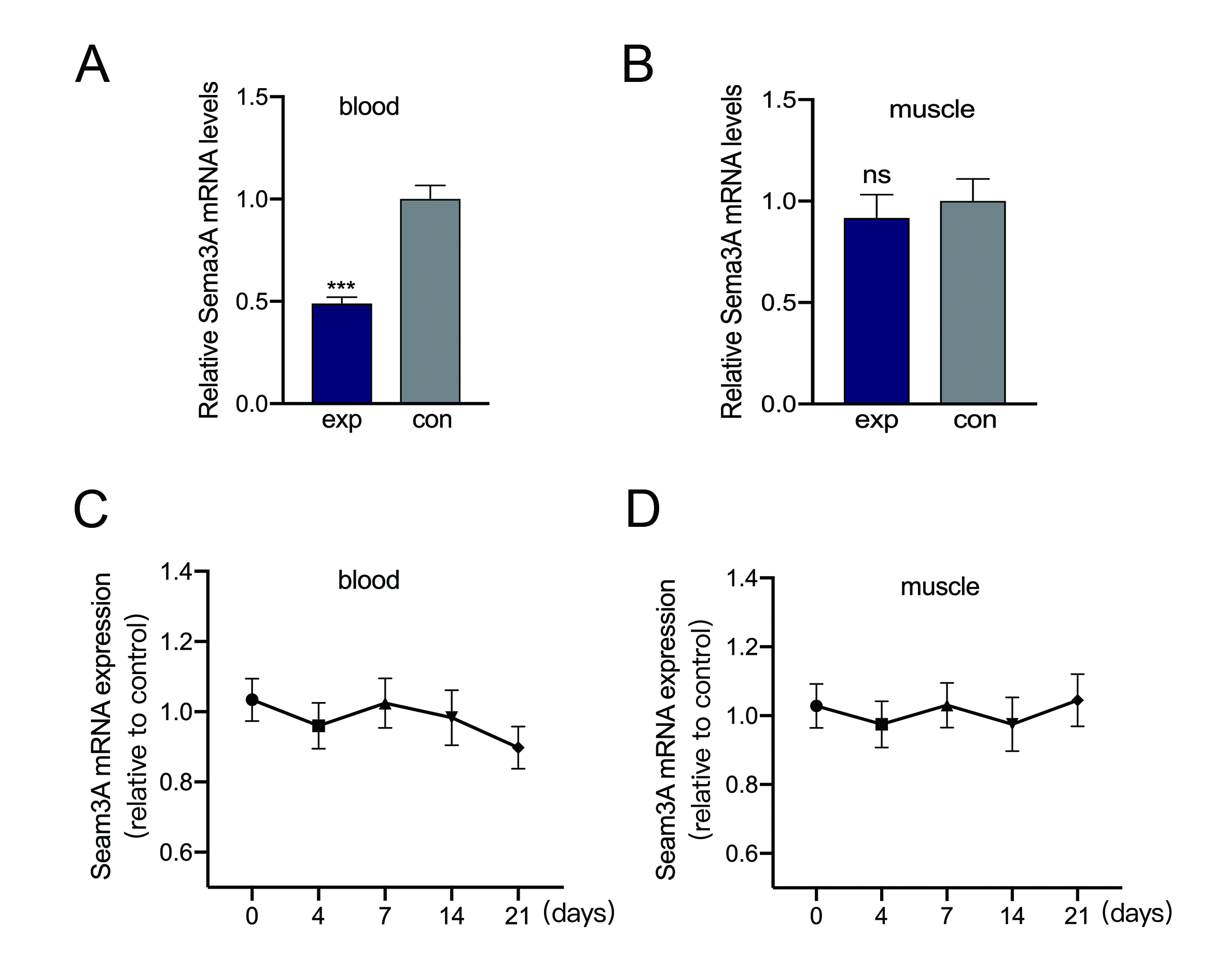

Supplement: Supplementary file 5 — Figure S3 [file 41418_2022_981_MOESM5_ESM.tif]

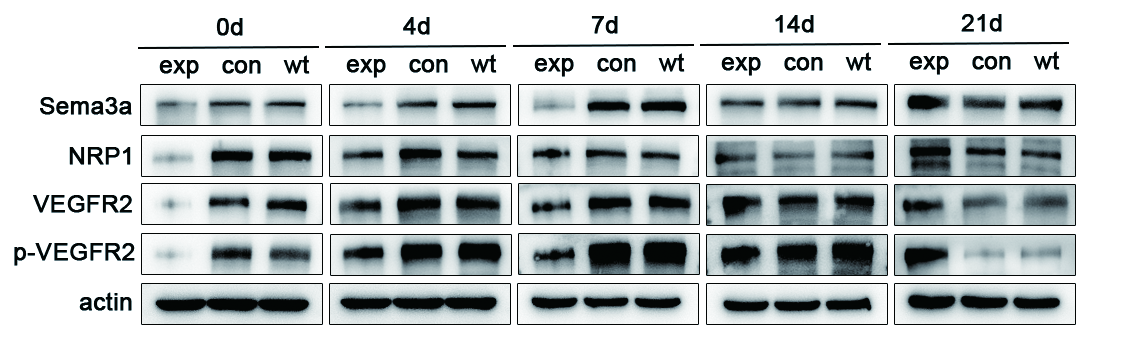

Supplement: Supplementary file 6 — Figure S4 [file 41418_2022_981_MOESM6_ESM.tif]

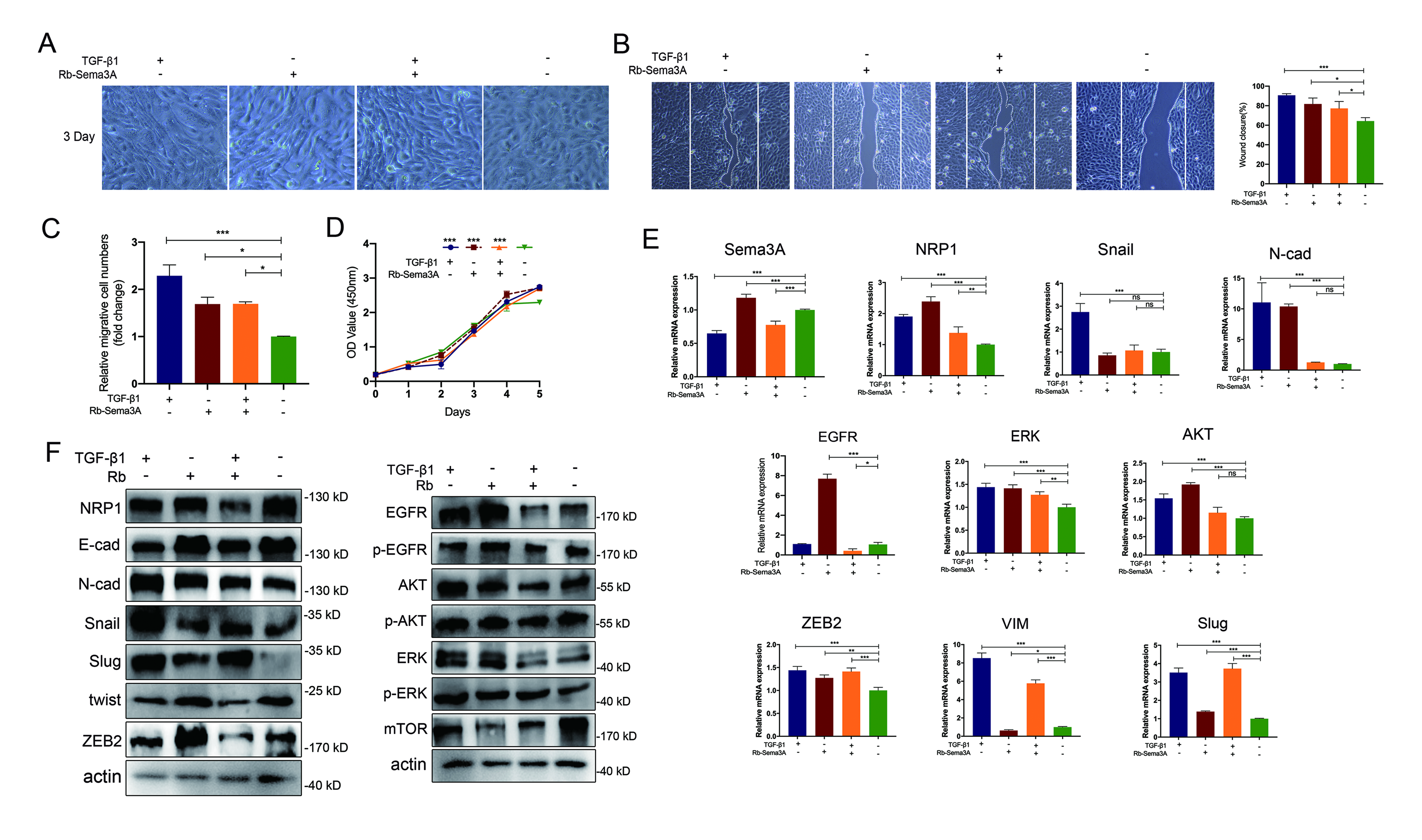

Supplement: Supplementary file 7 — Figure S5 [file 41418_2022_981_MOESM7_ESM.tif]

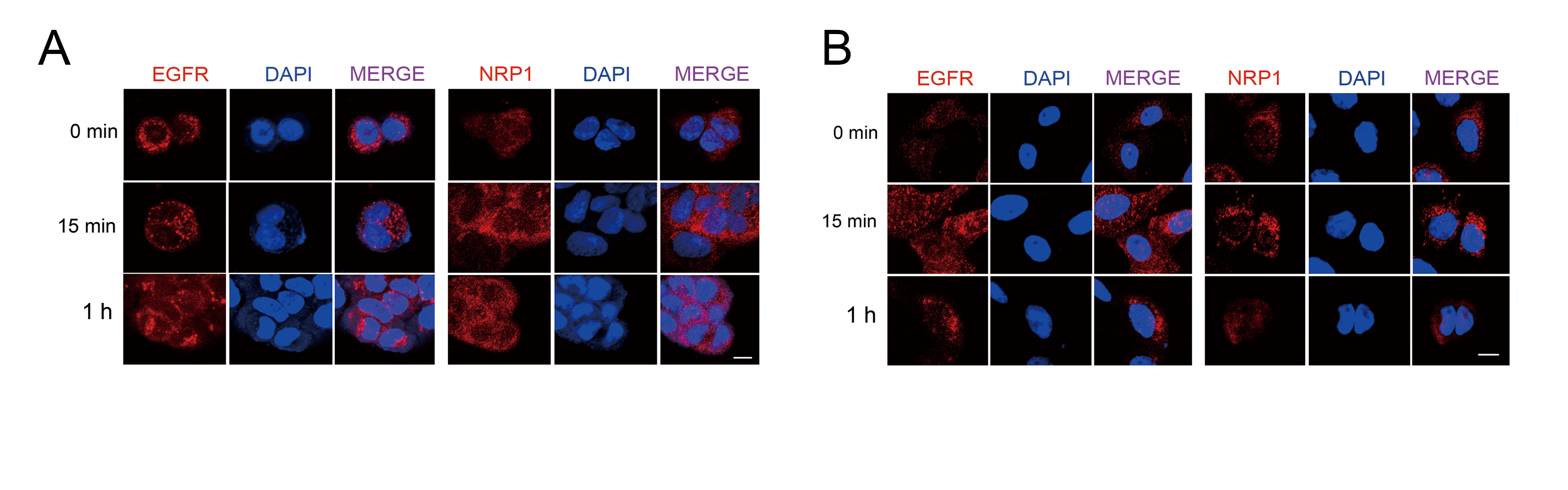

Supplement: Supplementary file 8 — Figure S6 [file 41418_2022_981_MOESM8_ESM.tif]

# Figure 1

G

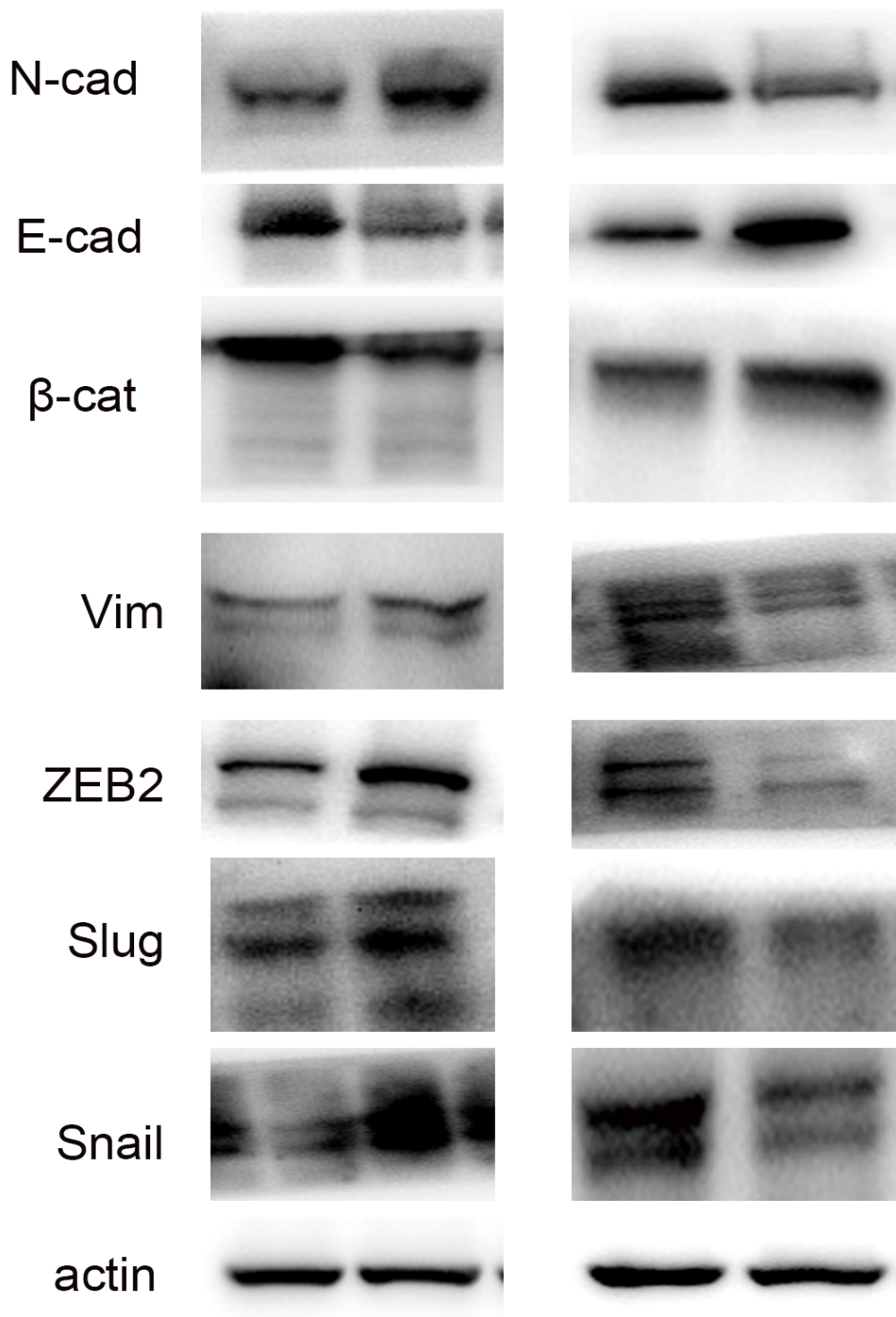

Figure 2

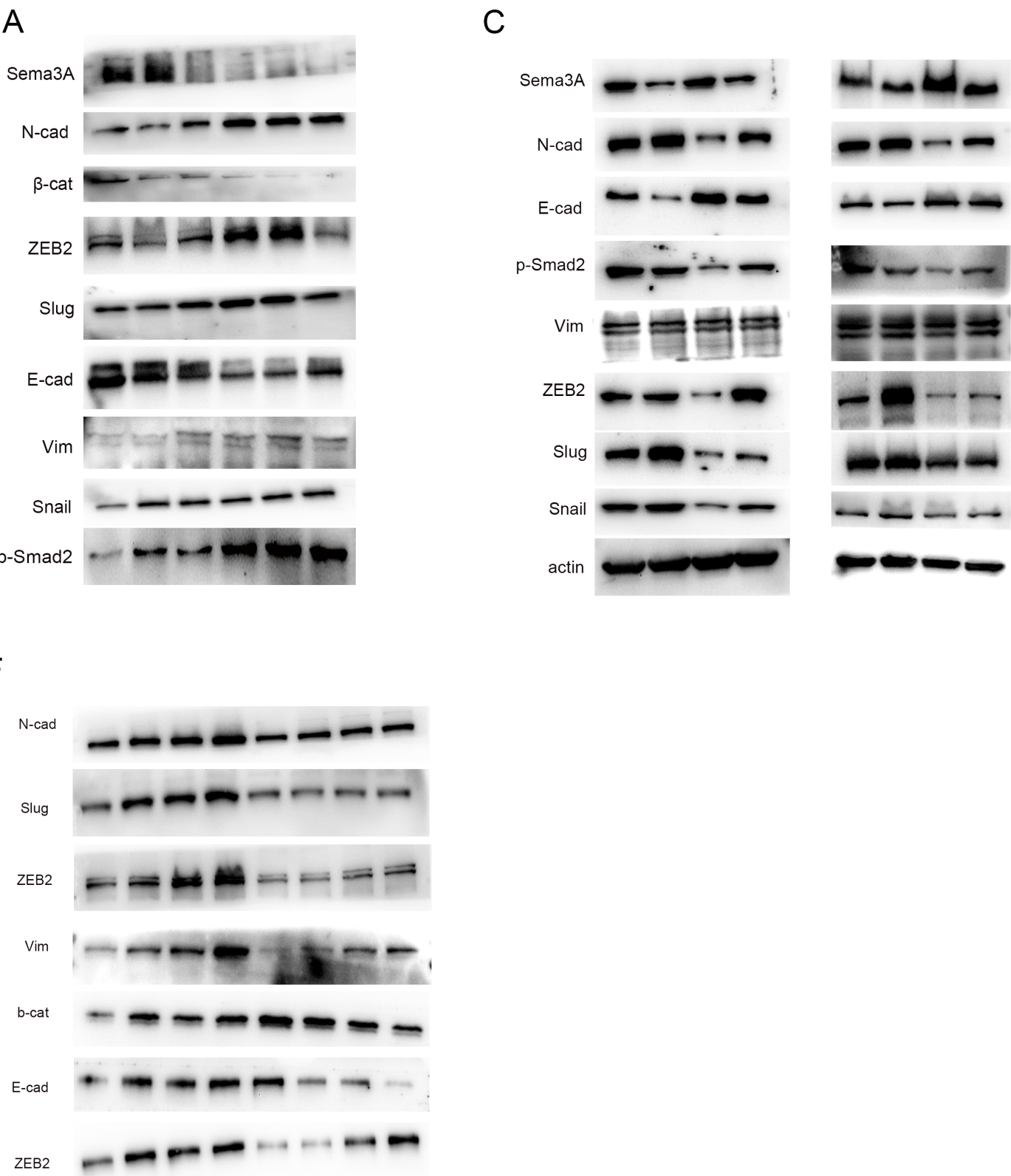

Figure 3

A

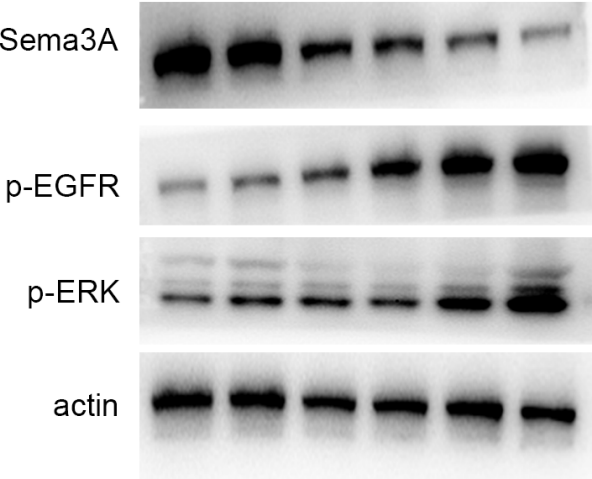

C

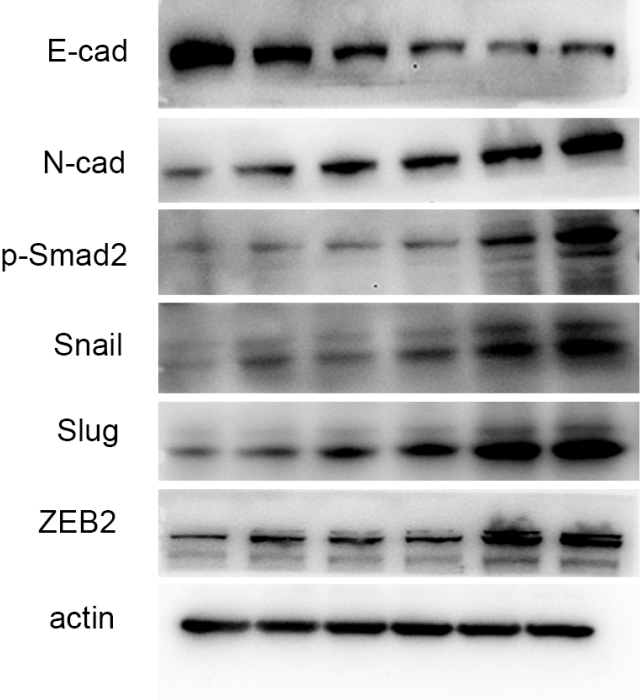

D

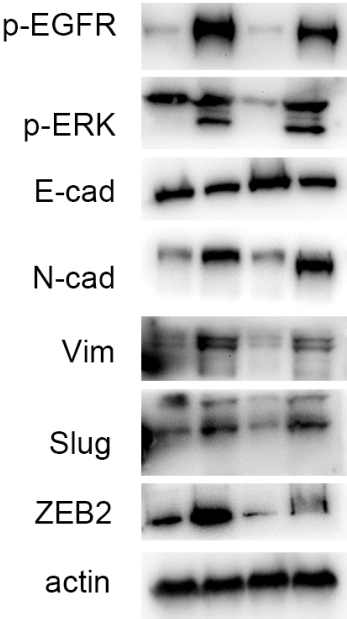

F

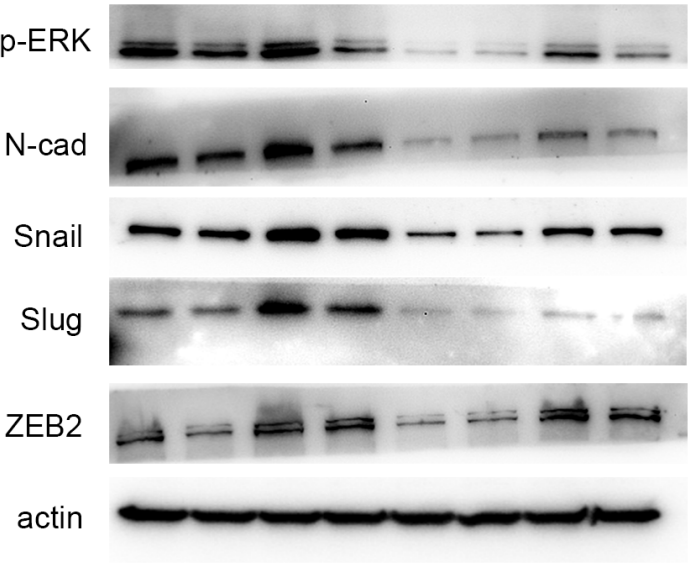

# Figure 5

E

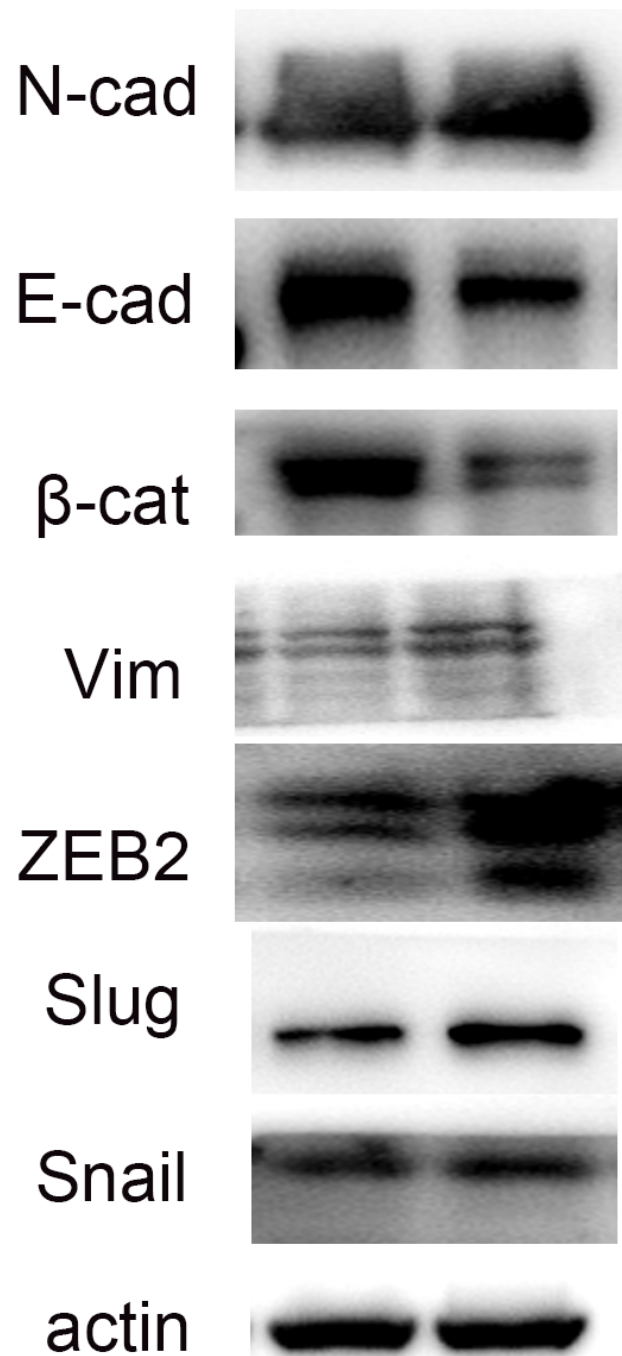

Figure 6

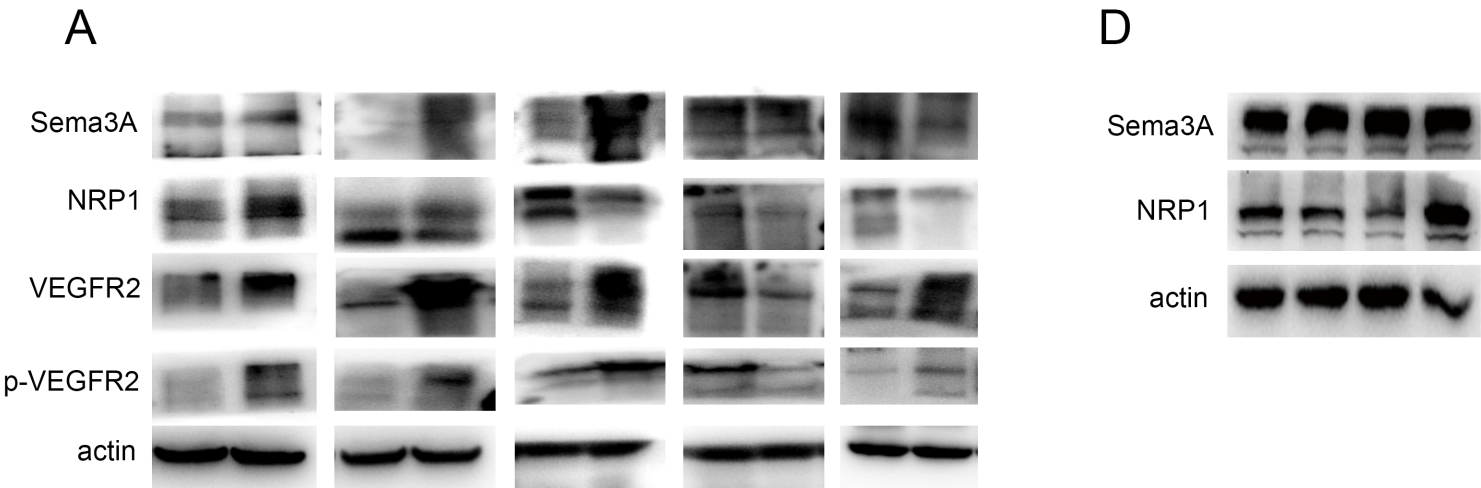

Figure 7

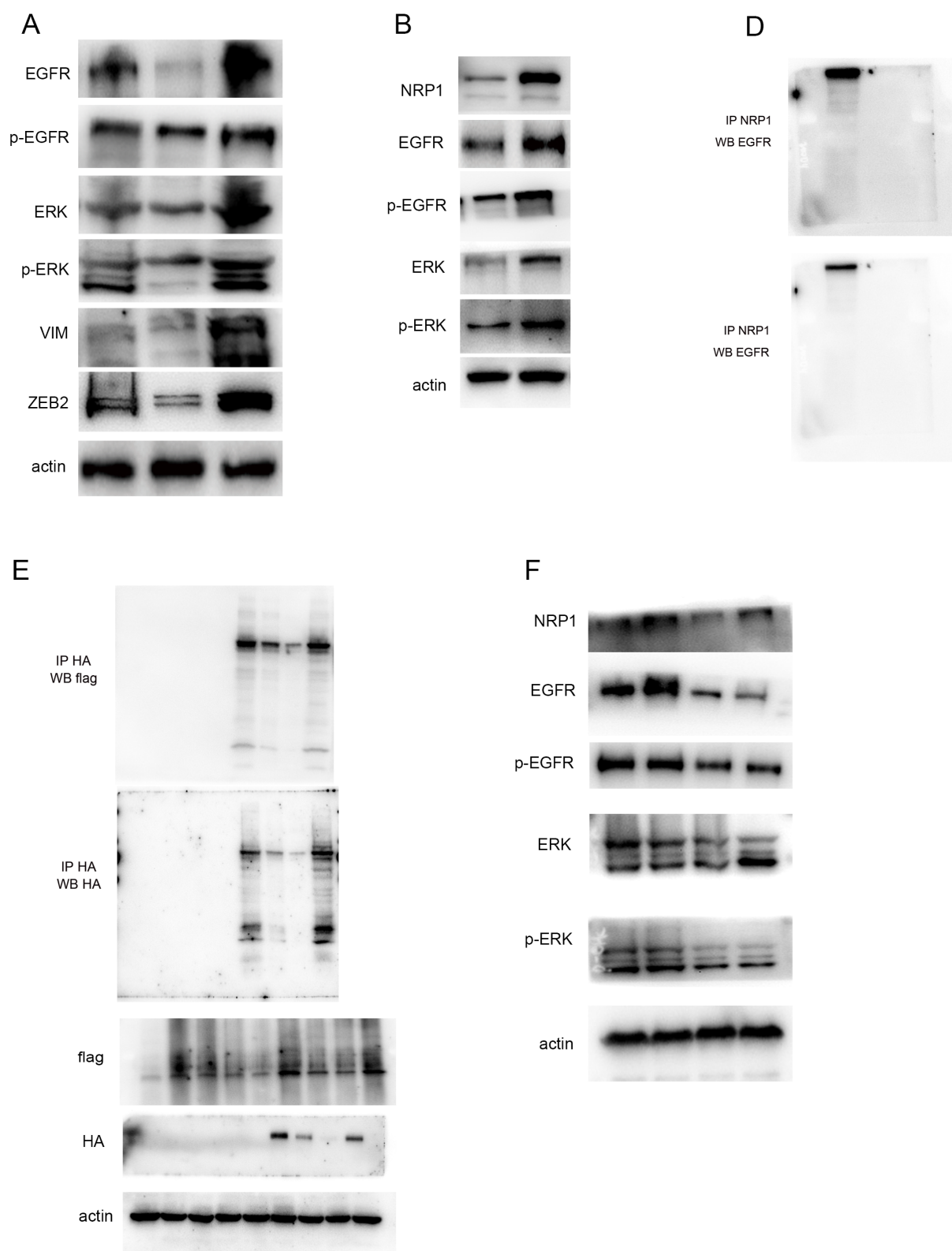

# Figure 8

# E

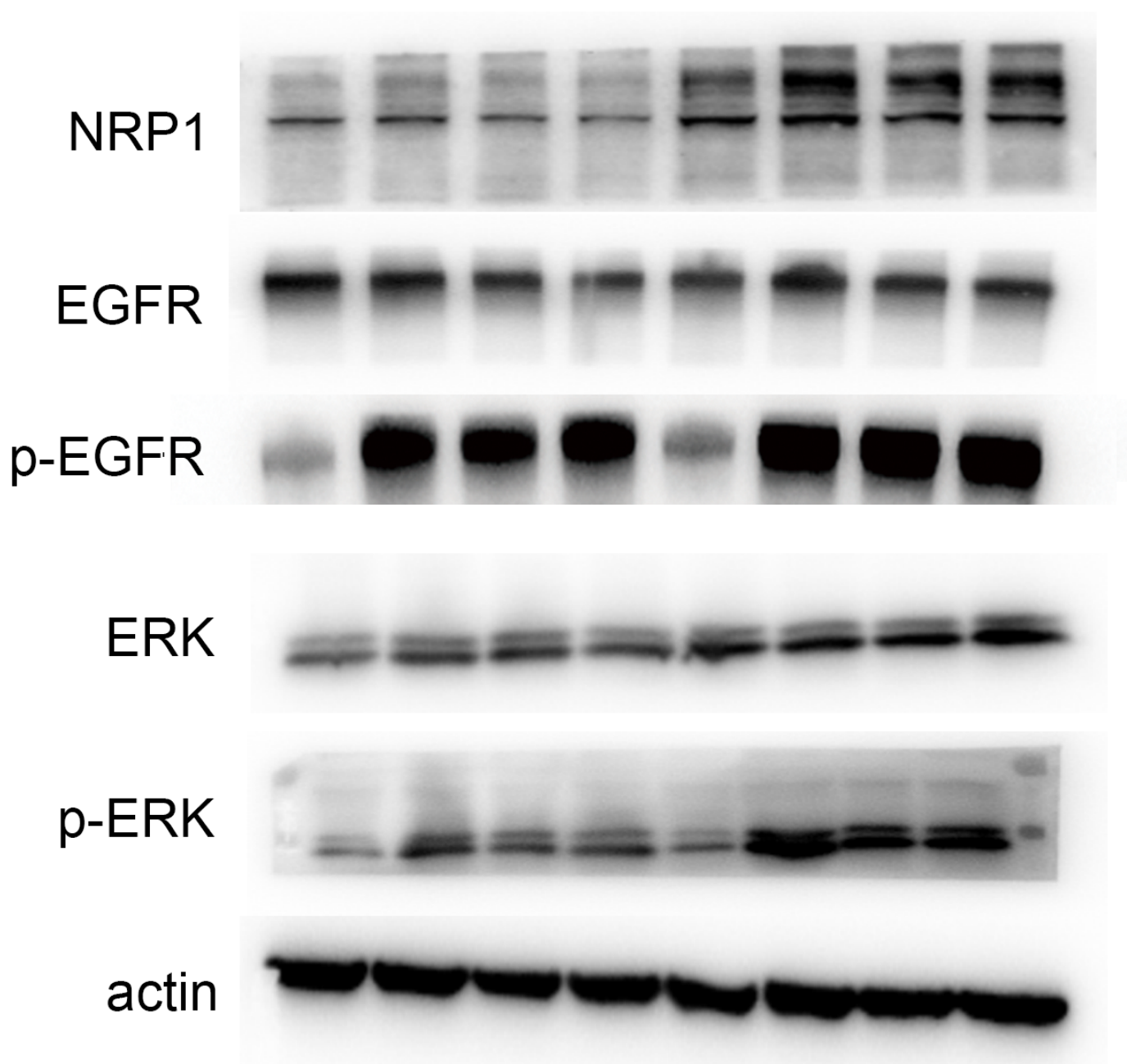

Supplement: Supplementary file 9 — Original Data for WB [file 41418_2022_981_MOESM9_ESM.pdf]
